# CARM1 methylates MED12 to regulate its RNA-binding ability

**DOI:** 10.26508/lsa.201800117

**Published:** 2018-09-19

**Authors:** Donghang Cheng, Vidyasiri Vemulapalli, Yue Lu, Jianjun Shen, Sayura Aoyagi, Christopher J Fry, Yanzhong Yang, Charles E Foulds, Fabio Stossi, Lindsey S Treviño, Michael A Mancini, Bert W O'Malley, Cheryl L Walker, Thomas G Boyer, Mark T Bedford

**Affiliations:** 1 Department of Epigenetics and Molecular Carcinogenesis, MD Anderson Cancer Center, The University of Texas, Smithville, TX, USA; 2 Cell Signaling Technology Inc., Danvers, MA, USA; 3 Department of Cancer Genetics and Epigenetics, Beckman Research Institute at City of Hope, Duarte, CA, USA; 4 Department of Molecular and Cellular Biology, Baylor College of Medicine, Houston, TX, USA; 5 Center for Precision Environmental Health, Baylor College of Medicine, Houston, TX, USA; 6 Department of Molecular Medicine, Institute of Biotechnology, University of Texas Health Science Center at San Antonio, San Antonio, TX, USA

## Abstract

The coactivator-associated arginine methyltransferase (CARM1) functions as a regulator of transcription by methylating a diverse array of substrates. To broaden our understanding of CARM1's mechanistic actions, we sought to identify additional substrates for this enzyme. To do this, we generated CARM1 substrate motif antibodies, and used immunoprecipitation coupled with mass spectrometry to identify cellular targets of CARM1, including mediator complex subunit 12 (MED12) and the lysine methyltransferase KMT2D. Both of these proteins are implicated in enhancer function. We identified the major CARM1-mediated MED12 methylation site as arginine 1899 (R^1899^), which interacts with the Tudor domain–containing effector molecule, TDRD3. Chromatin immunoprecipitation–seq studies revealed that CARM1 and the methyl mark it deposits are tightly associated with ERα-specific enhancers and positively modulate transcription of estrogen-regulated genes. In addition, we showed that the methylation of MED12, at the R^1899^ site, and the recruitment of TDRD3 by this methylated motif are critical for the ability of MED12 to interact with activating noncoding RNAs.

## Abstract (precis)

CARM1 methylates MED12 at arginine 1899 to generate a TDRD3 binding site, which in turn regulates the ability of mediator to interact with activating ncRNAs and modulate gene expression.

## Introduction

Arginine methylation is a prevalent posttranslational modification, and roughly 0.5% of arginine residues are methylated in MEFs (Dhar et al, 2013). This modification has been implicated in a myriad of biological processes such as transcription, splicing, signal transduction, and DNA repair (Yang & Bedford, 2013). Arginine methylation is catalyzed by a group of nine protein arginine methyltransferases (PRMTs), which can be classified into three types: type I (PRMT1, 2, 3, 4, 6, and 8) enzymes generate ω-*N*^*G*^*,N*^*G*^-asymmetric dimethylarginine (ADMA), type II (PRMT5 and 9) enzymes generate ω-*N*^*G*^*,N’*^*G*^-symmetric dimethylarginine (SDMA), and type III (PRMT7) enzyme forms ω-*N*^*G*^-monomethyl arginine residues in mammalian cells (Bedford & Clarke, 2009; Yang et al, 2015).

Coactivator-associated arginine methyltransferase (CARM1), which is also referred to as PRMT4, was first identified in a yeast two-hybrid screen for GRIP1-binding proteins (Chen et al, 1999). The recruitment of CARM1 to transcriptional promoters results in the methylation of the p160 coactivator family (SRC-1/NCOA1, SRC-2/GRIP1/NCOA2, and SRC-3/NCOA3), the histone acetyltransferases (p300/CREB-binding protein [CBP]), and the histone H3 (H3R17me2a and H3R26me2a) (Chen et al, 1999; Lee et al, 2005; Feng et al, 2006). These methylation events enhance gene activation; therefore, CARM1 is considered a secondary coactivator for nuclear receptor-mediated transcription. In addition, CARM1 was also shown to coactivate NF-κB (Covic et al, 2005). Furthermore, H3R17me2a chromatin immunoprecipitation (ChIP) studies showed elevated levels at a number of gene promoters including *pS2/TFF1*, *E2F1*, *CCNE1*, *aP2/FABP4*, *Oct4*, *Sox2*, *CITED2*, and *Scn3b* (Bauer et al, 2002; El Messaoudi et al, 2006; Frietze et al, 2008; Kleinschmidt et al, 2008; Yadav et al, 2008; Wu et al, 2009; Carascossa et al, 2010; O'Brien et al, 2010). CARM1 thus functions as a rather general transcriptional coactivator. Gene ablation studies in mice revealed that CARM1 is vital for existence (Yadav et al, 2003). Although CARM1 KO embryos are smaller in size, they are outwardly developmentally normal. These null embryos do display a number of cell differentiation defects, such as a partial block in T-cell development (Kim et al, 2004) and improper differentiation of lung alveolar cells (O'Brien et al, 2010) and adipocytes (Yadav et al, 2008). Enzyme-dead CARM1 knock-in mice phenocopy the null mice, indicating that CARM1's enzymatic activity is required for most of its in vivo functions (Kim et al, 2010). Thus, detailed knowledge of the spectrum of proteins that are methylated by CARM1 is critical for an in-depth understanding of how this enzyme regulates transcription in these different settings.

Substrate screening efforts by our laboratory and others have revealed two major classes of proteins that are methylated by CARM1: RNA-binding proteins and components of the transcriptional regulatory machinery. Large-scale enzyme reactions performed on high-density protein macroarrays led to the discovery of CARM1 substrates, such as the poly-A binding protein (PABP1) (Lee & Bedford, 2002). Using a small pool screening approach, splicing factors (SmB, SAP49, and U1C) and the transcription elongation factor (CA150) were identified as CARM1 substrates (Cheng et al, 2007). The RNA-binding proteins HuR and HuD (Li et al, 2002; Fujiwara et al, 2006) were identified by candidate approaches, as were other classes of proteins, including transcription factors (Sox2, Sox9, and Pax7) (Ito et al, 2009; Zhao et al, 2011; Kawabe et al, 2012), transcriptional coactivators including the SRCs and p300/CBP (Chen et al, 2000; Lee et al, 2005; Feng et al, 2006), and RNA Polymerase II C-terminal domain (Sims et al, 2011). Most PRMTs (PRMT1, 3, 5, 6, and 8) recognize and methylate a glycine and arginine-rich (GAR) motif (Yang & Bedford, 2013), which has facilitated the development of arginine methyl-specific antibodies that can be used to identify and help characterize substrates for this class of PRMTs. The first methyl-GAR motif antibodies (ADMA and SDMA) were developed by the Stéphane Richard laboratory and used in immunoprecipitation (IP)-coupled mass spectrometry (MS) experiments to identify novel methylated proteins (Boisvert et al, 2003). This approach has recently been expanded upon with the development of additional methyl-specific antibodies that recognize GAR-like monomethyl arginine and ADMA motifs using redundant peptide libraries with fixed methylarginine residues as antigens (Guo et al, 2014). An alternative (antibody-independent) approach to identifying methylated proteins is based on a modification of the “stable isotope labeling by amino acids in cell culture” (SILAC) technique, called heavy methyl SILAC (Ong et al, 2004). Heavy methyl SILAC exploits the fact that methionine is taken up by the cell and converted to the sole biological methyl donor, AdoMet. Thus, if [^13^CD_3_]methionine is used in these experiments, heavy methyl groups are incorporated into in vivo methylated proteins. Using this approach, in combination with methylarginine enrichment techniques, a large number of PRMT substrates were identified, including the mediator complex subunit 12 (MED12) (Uhlmann et al, 2012; Geoghegan et al, 2015). Subsequently, using ADMA antibodies, we identified MED12 as a heavily methylated protein (Guo et al, 2014), and Wei Xu's group further identified MED12 as a CARM1 substrate (Wang et al, 2015; Shishkova et al, 2017). Recently, the arginine demethylase JMJD6 was shown to interact with MED12 where it may counteract the activity of CARM1 (Gao et al, 2018).

Importantly, CARM1 has unique substrate specificity, and it does not methylate GAR motifs (Lee & Bedford, 2002; Cheng et al, 2007). Thus, to facilitate the rapid identification of new CARM1 substrates, we developed CARM1-motif antibody screening strategies to enrich for CARM1-methylated proteins. This approach is based on the finding that the H3R17me2a antibody (Millipore), which was raised against the asymmetric dimethyl arginine 17 mark on histone H3, cross-reacts with a number of CARM1 substrates when used for Western blotting protein extracts from WT and CARM1 KO embryos (Yadav et al, 2003; Cheng et al, 2012). These proteins were further confirmed to be CARM1 substrates (Cheng et al, 2007). Hence, we speculated that CARM1-methylated motifs have a rather loose consensus sequence and can be used to raise pan antibodies that would potentially recognize unknown CARM1 substrates. Using a cocktail of CARM1 methylated motifs as an antigen, we demonstrate that CARM1-motif antibodies can be developed. Furthermore, we identified KMT2D, G-protein pathway suppressor 2 (GPS2), and SLM2 as new CARM1 substrates, as well as a number of previously characterized CARM1 substrates, such as MED12.

Here, we confirmed that MED12 is methylated by CARM1 at R^1899^ in a nonredundant manner, and demonstrated that arginine methylation of MED12 positively regulates transcription of estrogen-regulated genes. To define the mechanistic basis for this effect, we found that the MED12-R^1899me2a^ mark serves as a docking site for the Tudor domain–containing effector molecule, TDRD3. TDRD3 is a known transcriptional coactivator that functions by recruiting topoisomerase (TOP3B) activity to chromatin (Yang et al, 2010, 2014). Importantly, we provide evidence that (1) CARM1 activity, (2) the R^1899^ methylation site on MED12, and (3) the recruitment of TDRD3 are all required for MED12 to bind activating long noncoding RNAs (ncRNAs).

## Results

### Developing and characterizing CARM1 substrate motif antibodies

**Figure 1.**
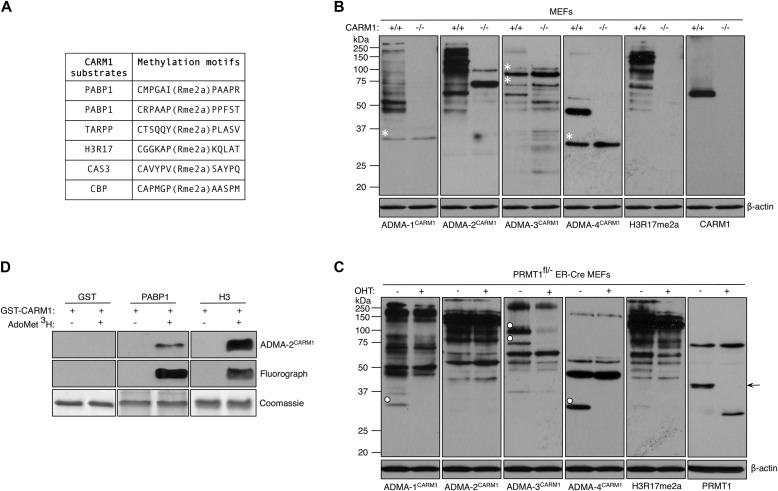
Characterization of CARM1 substrate antibodies. **(A)** The list of six peptides used to generate CARM1 substrate antibodies. **(B)** Whole cell extracts from CARM1 WT (+/+) and KO (−/−) MEFs were subjected to Western analysis with αADMA^CARM1^, αH3R17me2a, and αCARM1 antibodies. The asterisks on the gels indicate the positions of the proteins present in both cell lines. β-actin serves as a loading control. **(C)** PRMT1^fl/−^ ER-Cre MEFs were untreated or treated with 4-hydroxytamoxifen (OHT, 2 μM) for 8 d. Whole cell extracts were immunoblotted with αADMA^CARM1^, αH3R17me2a, and αPRMT1 antibodies. The solid circles on the gels indicate the positions of the proteins specific to PRMT1 WT (OHT−) MEFs. The arrow points to the position of the PRMT1 protein. β-actin serves as a loading control. **(D)** GST, PABP1, and H3 were methylated in vitro by recombinant CARM1 in the absence or presence of tritium-labeled AdoMet and subjected to Western analysis with αADMA-2^CARM1^ antibody (top panel), fluorography (middle panel), and Coomassie Brilliant Blue staining (bottom panel). GST serves as a negative control.

Methyl-specific antibodies (both ADMA and SDMA) have previously been used to identify PRMT substrates (Boisvert et al, 2003; Guo et al, 2014; Shishkova et al, 2017). These GAR-motif antibodies likely miss many of the CARM1 substrates because CARM1 cannot methylate these motifs. Indeed, the alignment of sites of known CARM1 methylation sites does not create a clear linear motif that could be used for in silico prediction of additional candidate substrates, although the motif appears proline-rich (Cheng et al, 2007; Shishkova et al, 2017). Thus, to facilitate the identification of CARM1 substrates, a peptide cocktail of six different CARM1-methylated motifs (Fig 1A) was used to immunize rabbits to obtain four CARM1 substrate motif antibodies, henceforth referred to as ADMA^CARM1^ antibodies. The four polyclonal antibodies were independently affinity purified over a column containing the six peptides.

To characterize the ADMA^CARM1^ antibodies, we performed Western analysis on whole cell lysates from WT and CARM1 KO MEFs (Fig 1B). ADMA-1^CARM1^ and ADMA-2^CARM1^ recognized multiple bands in the WT lane, but displayed minimal immunoreactivity in the KO lane. ADMA-3^CARM1^ showed the loss of one band but also a slightly increased signal in the KO lane relative to the WT lane, suggesting that it might recognize proteins methylated by CARM1 and other type I PRMTs, compensating the loss of CARM1 in KO cells. ADMA-4^CARM1^ seemed to be less immunoreactive than the other three antibodies, recognizing only a single CARM1 substrate. As anticipated, the H3R17me2a antibody cross-reacted with a number of proteins in WT cells, which are absent in CARM1 KO cells. Interestingly, some proteins were recognized in WT and KO cells at similar levels (indicated by asterisks). Next, we tested the antibodies on WT and PRMT1-deficient MEFs. Most proteins were recognized at similar levels in both cell lines. However, some protein bands were specific to WT cells, not PRMT1-deficient cells (indicated by solid circles) (Fig 1C). It is noteworthy that these PRMT1-specific proteins migrated at the same positions as the nonspecific proteins observed in CARM1 MEFs (asterisks in Fig 1B). This suggests that although the ADMA^CARM1^ antibodies primarily recognize CARM1 substrates, they can also recognize a few PRMT1 substrates.

We next tested the ability of some of the ADMA^CARM1^ antibodies to recognize in vitro methylated CARM1 substrates. PABP1 and H3 were in vitro methylated by CARM1 in the absence or presence of tritium-labeled AdoMet. Methylation efficiency was monitored by fluorography (Fig 1D, middle panel), and duplicate blots were subjected to Western blotting using the ADMA^CARM1^ antibodies. We found that the ADMA-2^CARM1^ antibody recognized PABP1 and H3 in a methyl-specific fashion (Fig 1D, top panel). These results confirm the establishment of a panel of four distinct rabbit polyclonal antibodies that recognize different subsets of CARM1 substrates.

### Using ADMA^CARM1^ antibodies to identify CARM1 substrates

**Figure 2.**
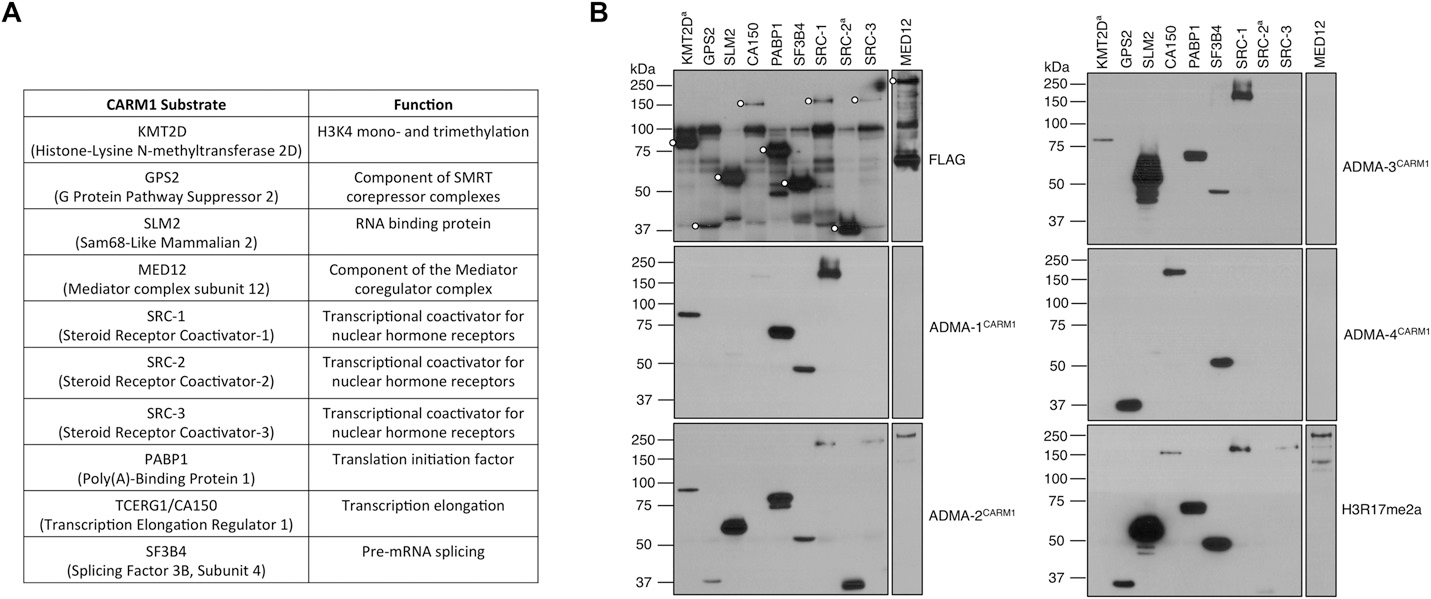
Identification of novel CARM1 substrates. **(A)** A table showing the partial list of CARM1 substrates, identified from the αADMA^CARM1^ IP-MS screen, and their functions. **(B)** The positive hits obtained from the screen were transiently transfected into HEK293T cells and immunoprecipitated with αFLAG antibody. Western analysis was performed, first with αFLAG to gauge the expression of the FLAG-tagged proteins (indicated by solid circles) and then with the αADMA^CARM1^ antibodies. KMT2D^a^ (3,619–4,285 aa) and SRC-2^a^ (1,037–1,295 aa) represent fragments of the full-length KMT2D and SRC-2 proteins.

A combined IP using a cocktail of the four ADMA^CARM1^ antibodies and MS approach was used to identify CARM1 substrates, using an approach developed for the identification of tyrosine phosphorylation sites (Rush et al, 2005), and 112 different proteins were identified as putative CARM1 substrates. All the identified methylation sites have been submitted to the PhosphoSitePlus database (www.phosphosite.org). We selected 10 proteins for further evaluation based on the number of identified methylated peptides in the MS data, and the potential involvement of the candidate CARM1 substrates in different aspects of chromatin regulation, transcription, and RNA processing (Fig 2A). Of the 10 identified substrates, PABP1 (Lee & Bedford, 2002), CA150/TCERG1 (Cheng et al, 2007), and SRC-3 (Feng et al, 2006) were described previously as in vivo CARM1 substrates, thus validating the approach. SF3B4 (Cheng et al, 2007), MED12 (Shishkova et al, 2017), SRC-1 (Feng et al, 2006), and SRC-2 (Feng et al, 2006) were previously shown to be methylated by CARM1 in vitro. GPS2, KMT2D, and SLM2 were identified as potential novel CARM1 substrates in this study. MED12 is a subunit of the mediator complex, which functions in relaying regulatory signals from transcription factor–bound enhancers to RNA Pol II (Allen & Taatjes, 2015), and seems to be a major target for CARM1 methylation (Shishkova et al, 2017). GPS2 is an integral component of the NCoR/SMRT/HDAC3 corepressor complex (Wong et al, 2014). The mixed-lineage leukemia 4 methyltransferase (KMT2D) is responsible for depositing histone H3K4me1/2 marks at enhancers (Lee et al, 2013; Ford & Dingwall, 2015). SLM2 is a KH domain containing protein that has been implicated in the regulation of alternative splicing (Traunmuller et al, 2014).

To confirm that the identified proteins were indeed recognized by the methyl-specific antibodies that we developed and used in the screen, we FLAG-tagged the 10 selected proteins, overexpressed them in HEK293T cells, and then immunoprecipitated them using an anti-FLAG antibody. Western analysis was performed on the immunoprecipitates, first with an anti-FLAG antibody to confirm the expression of FLAG-tagged proteins, and then with the ADMA^CARM1^ antibodies (Fig 2B). Importantly, all 10 proteins were recognized by at least one of the four different ADMA^CARM1^ antibodies, and no single antibody recognized all the tagged proteins. Thus, none of the four ADMA^CARM1^ antibodies are totally pan CARM1 substrate antibodies, and they clearly recognize different subsets of CARM1 substrates.

**Figure S1.**
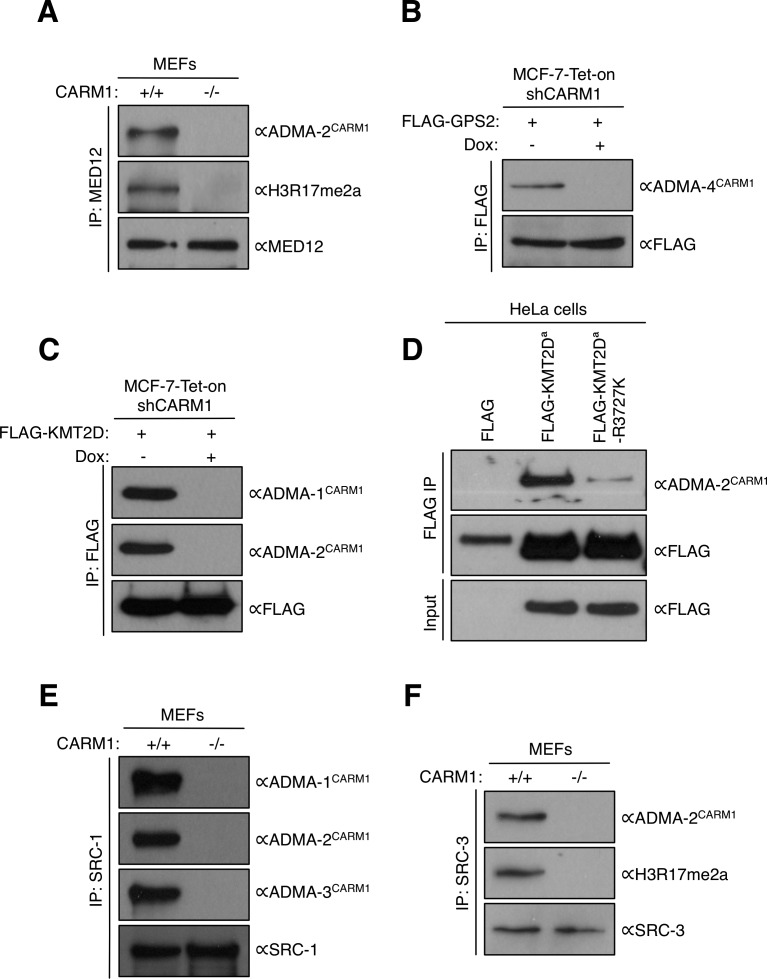
Validation of novel CARM1 substrates. **(A, E, F)** Whole cell lysates from CARM1 WT (+/+) and KO (−/−) MEFs were immunoprecipitated with antibodies against MED12, SRC-1, or SRC-3 proteins, and the eluted samples were subjected to Western blotting with αADMA^CARM1^ and αH3R17me2a antibodies. αMED12, αSRC-1, and αSRC-3 blots (bottom panels in A, E, F) demonstrate equal expression of these proteins in CARM1 WT (+/+) and KO (−/−) MEFs. **(B, C)** MCF-7-Tet-on-shCARM1 cells were untreated or treated with doxycycline (1 μg/ml) for 8 d and transiently transfected with FLAG-KMT2D and -GPS2, separately. Total cell lysates were immunoprecipitated with αFLAG antibody, and the eluted samples were subjected to Western blotting with αADMA^CARM1^ and αFLAG antibodies. **(D)** HeLa cells were transiently transfected with FLAG control, FLAG-KMT2D^a^, or FLAG-KMT2D^a^-R3727K. Total cell lysates were immunoprecipitated with αFLAG antibody, and the eluted samples were subjected to Western analysis with αADMA^CARM1^ and αFLAG antibodies. αFLAG blot of the input samples shows equal expression of the WT and mutant KMT2D^a^ proteins. KMT2D^a^ represents a fragment (3,619–4,285 aa) of the full-length KMT2D protein (NP_003473).

In Fig 1B, we show that the ADMA^CARM1^ antibodies primarily recognize CARM1 substrates, but they are also able to engage a few PRMT1 substrates (Fig 1C). To be sure that the new methylated proteins that we identified are indeed CARM1 and not PRMT1 substrates, we performed further analysis in CARM1 KO and knock-down cell lines that we had previously established (Yadav et al, 2003; Yang et al, 2010). We immunoprecipitated endogenous MED12 from CARM1 WT and KO MEFs, and then performed Western blot analysis using ADMA^CARM1^ (and αH3R17me2a) antibodies (Fig S1A). Both cell lines had the same amount of MED12, but only MED12 isolated from CARM1 expressing cells was immunoreactive with the two CARM1 substrate motif antibodies. These data indicate that arginine methylation of MED12 is CARM1-dependent. GPS2, KMT2D, and SLM2 were not tested in this assay, because we were unable to find specific antibodies that were able to immunoprecipitate the respective endogenous proteins. We thus overexpressed FLAG-GPS2 in control and CARM1-knockdown cells, and then performed Western blotting of the FLAG immunoprecipitates using ADMA^CARM1^ antibodies to show that GPS2 is specifically recognized in control cells but not in CARM1-knockdown cells (Fig S1B). In the case of KMT2D, owing to its large size (5,537 aa), we cloned a fragment (3,619–4,285 aa) of the full-length KMT2D protein, which harbors the MS identified methylation sites (R3727 [major] and R4212 [minor]). We also engineered a R3727K mutation at this site in the FLAG-KMT2D^a^ construct, and found that the ADMA^CARM1^ signal is significantly reduced in the R3727K-KMT2D^a^ mutant, relative to the WT KMT2D^a^ protein, indicating that R3727 is a major site for CARM1 methylation (Fig S1C and D). Finally, we also confirmed that steroid receptor coactivators SRC-1 (Fig S1E) and SRC-3 (Fig S1F) are recognized in a CARM-dependent fashion by the ADMA^CARM1^ antibodies.

### Mediator subunit 12 is methylated at Arginine 1899 by CARM1 in vivo

**Figure 3.**
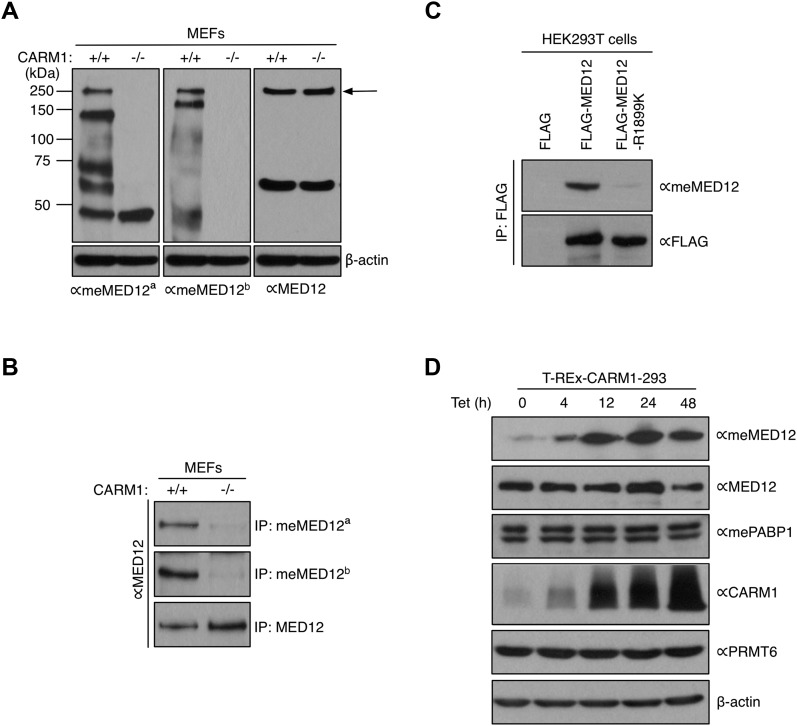
Characterization of two meMED12 antibodies. **(A)** Whole cell extracts from CARM1 WT (+/+) and KO (−/−) MEFs were subjected to Western analysis with αmeMED12^a^, αmeMED12^b^, and αMED12 antibodies. The arrow points to the position of the MED12 protein. β-actin serves as a loading control. **(B)** CARM1 WT (+/+) and KO (−/−) MEFs were immunoprecipitated with αmeMED12^a^, αmeMED12^b^, and αMED12 antibodies, and the eluted samples were subjected to Western blotting with αMED12 antibody. **(C)** HEK293T cells were transiently transfected with FLAG, FLAG-MED12, or FLAG-MED12-R1899K (NP_005111). Total cell lysates were immunoprecipitated with αFLAG antibody and the eluted samples were subjected to Western analysis with αmeMED12^a^ and αFLAG antibodies. **(D)** T-REx-CARM1-293 cells were treated with tetracycline and harvested after 0, 4, 12, 24, and 48 h. Whole cell extracts were immunoblotted with the indicated antibodies. β-actin serves as a loading control.

The MS studies identified MED12-R1899 as a major site of CARM1 methylation. To characterize the methylation site in more detail, we raised two independent antibodies against the MED12-R1899me2a peptide motif. Western blot analysis of CARM1 WT and KO MEF extracts showed that both antibodies detect a 240 kD protein (the expected size of MED12) and a few other potential substrates, in a CARM1-dependent manner (Fig 3A). The meMED12^b^ antibody was more selective (only recognized one additional band of about 200 kD) and was used for most of the following studies. To confirm that the 240 kD protein is MED12, we immunoprecipitated methylated MED12 from WT and CARM1 KO MEF lysates with the meMED12 antibodies and blotted with the MED12 (Bethyl) antibody. We found that both meMED12 antibodies selectively enriched MED12 protein from WT cells (Fig 3B). To confirm that the meMED12 antibodies are site-specific, we made a R1899K point mutation in the full-length FLAG-MED12 construct, immunoprecipitated the WT and mutant forms from transiently expressing HEK293T cells, and Western blotted with meMED12 antibody. The antibody detected the WT form of FLAG-MED12 strongly, but not the R1899K mutant form (Fig 3C). All these data clearly show that MED12 is methylated by CARM1 at R1899.

**Figure S2.**
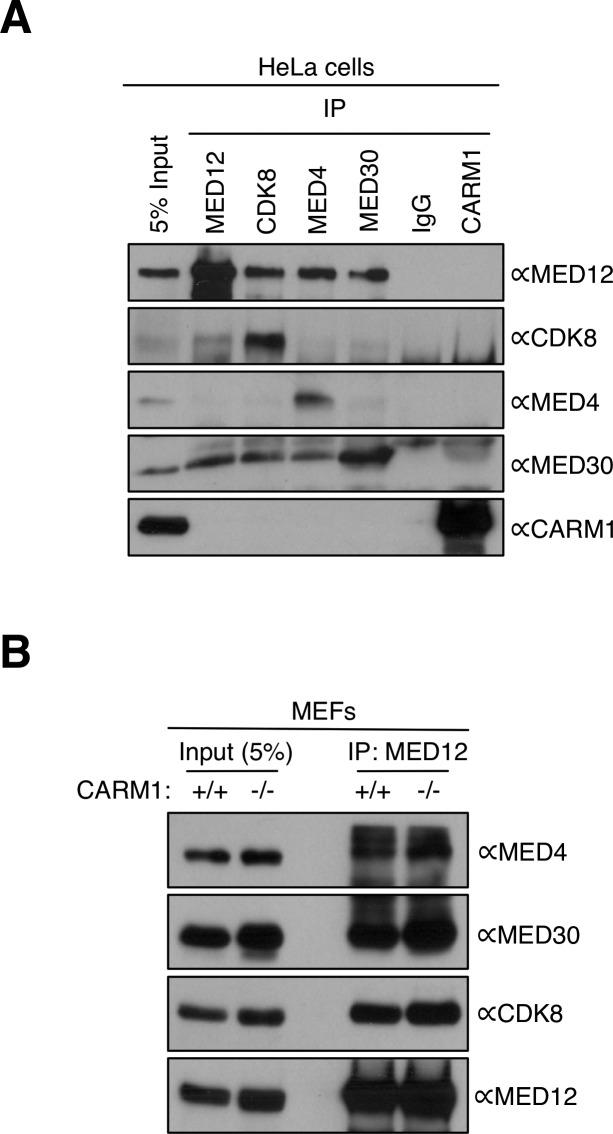
CARM1 and mediator interactions. **(A)** CARM1 does not directly associate with the mediator complex. HeLa whole cell lysates were subjected to IP using IgG or antibodies specific for MED12, CDK8, MED4, MED30, and CARM1. Western blotting was performed on the immunoprecipitates using antibodies against CARM1 or the indicated mediator subunits. **(B)** Methylation of MED12 does not affect mediator complex assembly. Whole cell extracts from CARM1 WT (+/+) and KO (−/−) MEFs were immunoprecipitated with αMED12 antibody and subjected to Western analysis using antibodies specific for the indicated mediator subunits.

CARM1 occasionally stably interacts with its substrates (Feng et al, 2006; Kowenz-Leutz et al, 2010; Wang et al, 2014). To determine if CARM1 stably associates with the mediator complex, we performed reciprocal co-IP experiments between CARM1 and mediator subunits (MED12, MED4, MED30, and CDK8) from HeLa cell lysates. We found that CARM1 does not stably interact with the mediator complex (Fig S2A). Thus, CARM1 and MED12 are likely transiently engaged. We also tested if MED12 methylation has a role in mediator complex assembly. Co-IPs were performed between MED12 and other mediator subunits in WT and CARM1 KO MEFs. We found that the MED12 antibody co-immunoprecipitated mediator subunits (MED4, MED30, and CDK8) from WT and KO cells at similar levels. This suggests that MED12 is incorporated into the mediator complex, irrespective of its methylation status (Fig S2B).

Arginine methylated proteins, PABP1, and SAP145, are thought to be fully methylated in cells (Zeng et al, 2013; Yang et al, 2015), suggesting that these are not “regulatable” signaling nodes. To test if MED12 is fully methylated in cells, we used the tetracycline-inducible CARM1-Flp-In HEK293 cell system (Cheng et al, 2007). Upon the induction of CARM1 expression, we observed an increase in MED12 methylation levels, as detected by the meMED12 antibody (Fig 3D). On the other hand, methylated PABP1 levels did not change with CARM1 induction, as expected. Therefore, MED12 is not fully methylated in cells, and the dynamic changes in methylation levels upon CARM1 over-expression point toward a regulatory role for this R1899 methylation. Thus, transient association of CARM1 with MED12 at enhancer elements may induce local methylation of the R1899 site to facilitate the docking of a “reader” molecule.

### Methylated MED12 interacts with the effector molecule TDRD3

**Figure 4.**
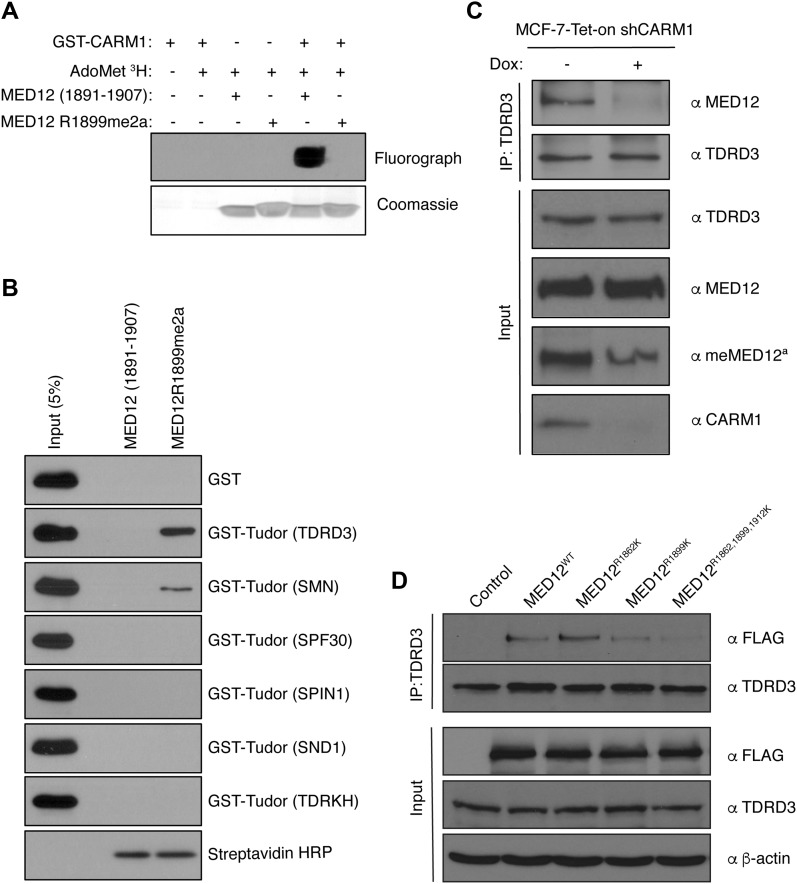
MED12 interacts with TDRD3 in a CARM1-dependent fashion. **(A)** Fluorograph (top panel) and Coomassie Brilliant Blue staining (bottom panel) of the peptides in vitro methylated by recombinant CARM1 in the presence of tritium-labeled AdoMet. **(B)** The peptides were used to pull down Tudor domains of the indicated proteins. The input samples and the eluted samples were immunoblotted with αGST antibody. Streptavidin HRP serves as a peptide loading control. **(C)** MCF-7-Tet-on-shCARM1 cells were untreated or treated with doxycycline (1 μg/ml) for 8 d. Nuclear extracts were subjected to IP with αTDRD3 antibody and the eluted samples were detected by Western blotting with αMED12 and αTDRD3. The input samples were immunoblotted with αTDRD3, αMED12, αmeMED12^a^, and αCARM1. **(D)** HEK293T cells were transiently transfected with FLAG, FLAG-MED12 WT, FLAG-MED12-R1862K, FLAG-MED12-R1899K, and FLAG-MED12-R1862,1899,1912K. Total cell lysates were immunoprecipitated with αTDRD3 antibody and the eluted samples were subjected to Western analysis with αFLAG and αTDRD3 antibodies. The input samples were immunoblotted with αFLAG, αTDRD3, and αβ-actin.

Effector molecules for both ADMA and SDMA motifs are Tudor domain–containing proteins (Cote & Richard, 2005; Gayatri & Bedford, 2014). For example, the Tudor domain of SMN binds spliceosomal proteins such as SmB (Brahms et al, 2001; Friesen et al, 2001) and SAP145 (Yang et al, 2015), and TDRD3 was shown to bind the CARM1 histone code mark H3R17me2a (Yang et al, 2010). To determine if the methylated R1899 motif of MED12 interacts with any of the known methylarginine “reading” Tudor domain–containing proteins, we synthesized biotin-tagged, unmodified or methylated, MED12 peptides and validated them by an in vitro methylation assay. As expected, recombinant CARM1 methylated the unmodified MED12 peptide in vitro, but not the methylated peptide, which has no methyl-acceptor position (Fig 4A). This experiment also independently confirms that the MED12 R1899 site is indeed a CARM1 methylation motif. These peptides were then used to pull down GST-fused Tudor domains of TDRD3, SMN, SPF30, TDRKH, SPIN1, and SND1, the six best-characterized methylarginine-“reading” Tudor domain–containing proteins (Gayatri & Bedford, 2014). Pull-down experiments demonstrated that the Tudor domain of TDRD3 bound strongly to the methylated form of the MED12 peptide, whereas SMN bound weakly (Fig 4B). Next, we endeavored to confirm that MED12 interacts with TDRD3 in cells, and that this interaction is CARM1-dependent. To do this, we used CARM-inducible knockdown cells, and immunoprecipitated TDRD3 from CARM1 WT and KD cells. TDRD3 co-immunoprecipitated MED12 from WT, but not KD cells (Fig 4C). Input controls show that CARM1 was efficiently knocked-down and that MED12 methylation levels were decreased. These data establish that TDRD3 interacts with MED12 in a CARM1-dependent manner. MED12 was previously shown to be methylated by CARM1 at two additional sites (R1862 and R1912) (Wang et al, 2015). To establish the importance of all three methylation sites (R1862, R1899, and R1912) in driving the MED12-TDRD3 interactions, we transiently transfected HEK293T cells with FLAG-tagged full-length MED12, and its corresponding mutants and immunoprecipitated endogenous TDRD3 (Fig 4D). These co-IP experiments revealed that the R1862 and R1912 are not critical for this interaction, but that the R1899 site is important.

### Genome-wide and H3R17me2a chromatin occupancy

**Figure 5.**
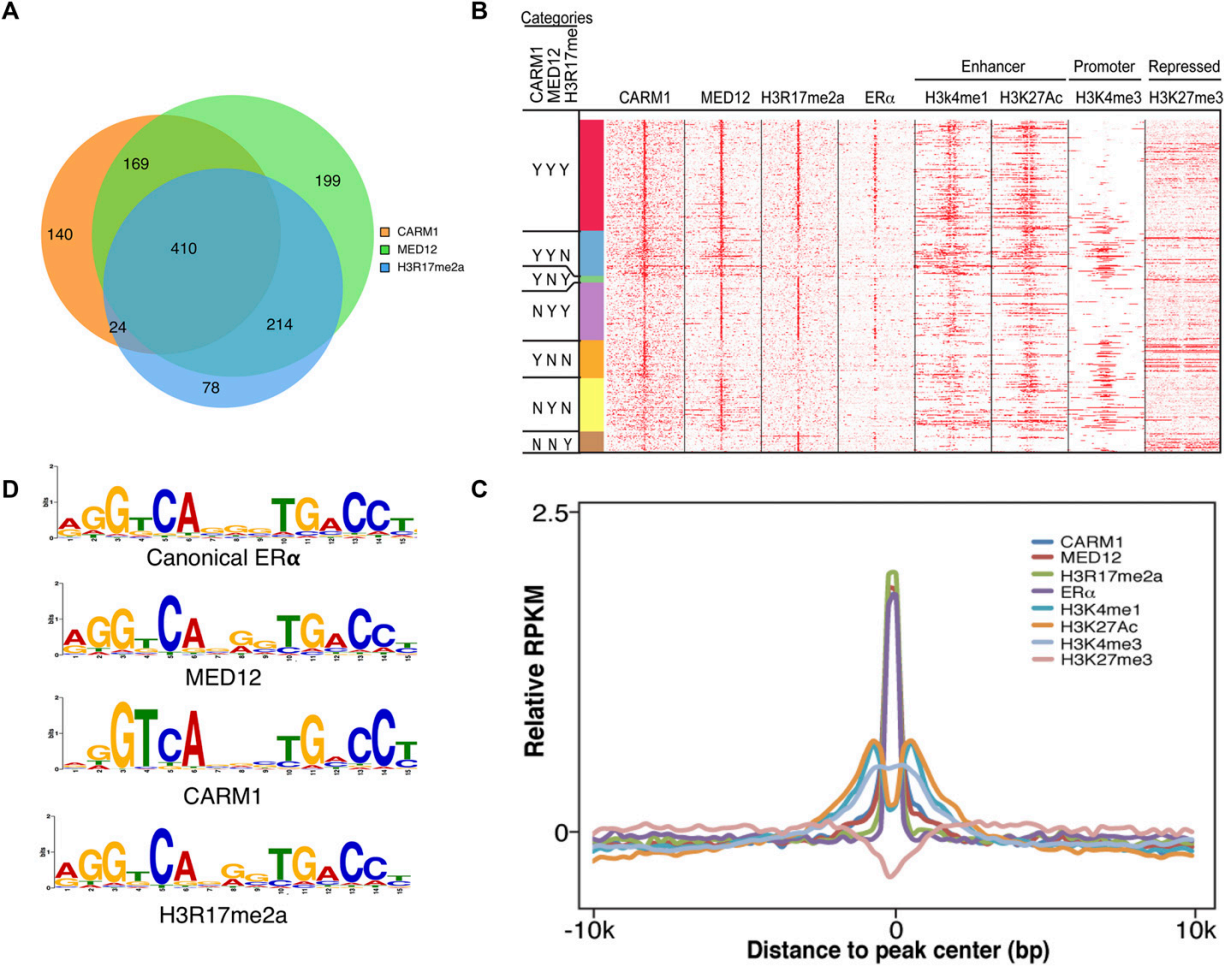
ChIP-seq analysis of CARM1, MED12, and H3R17me2a in MCF-7 cells. **(A)** Venn diagram showing an overlap between CARM1-, MED12-, and H3R17me2a-binding sites on the MCF-7 genome. **(B, C)** Heatmap and distribution figures depict profiles for genome-wide localization of CARM1, MED12, H3R17me2a, ERα, enhancer (H3K4me1 and H3K27ac), promoter (H3K4me3), and repressor (H3K27me3) marks. Categories: YYY—peaks co-occupied by CARM1, MED12, and H3R17me2a; YYN—peaks co-occupied by CARM1 and MED12; YNY—peaks co-occupied by CARM1 and H3R17me2a; NYY—peaks co-occupied by MED12 and H3R17me2a; YNN, NYN, and NNY—peaks occupied by CARM1, MED12, and H3R17me2a, respectively. **(D)** Comparison of the binding motifs for MED12, CARM1, H3R17me2a, and ERα.

**Figure S3.**
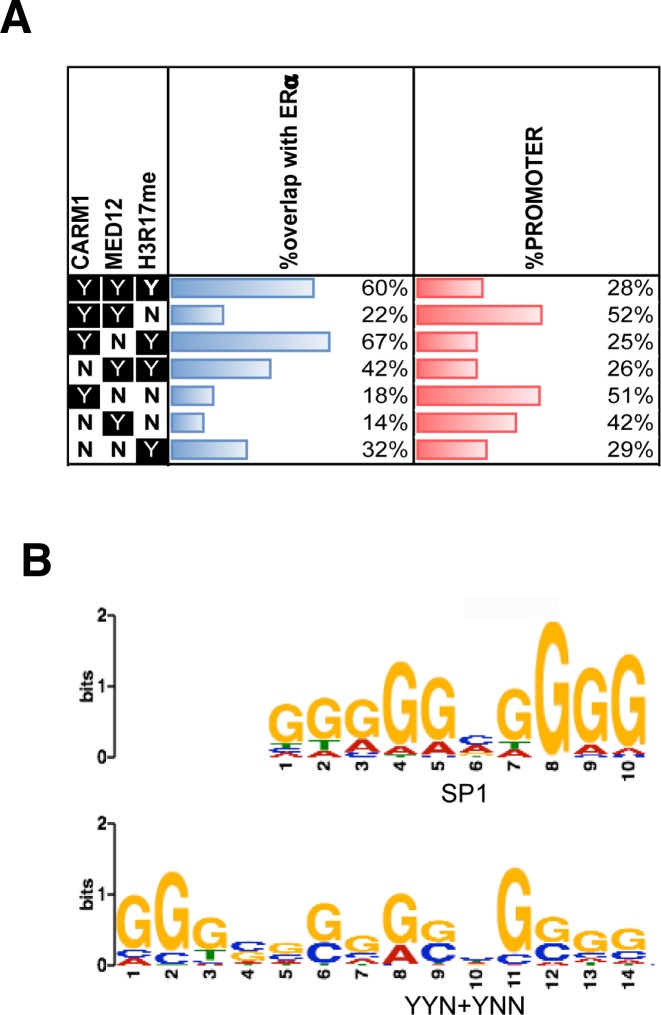
ChIP-seq peak analysis. **(A)** Percentage of peaks overlapping ERα binding sites and percentage of peaks in promoter; ERα peaks were called by MACS at *P-*value 1 × 10^−10^. Promoter was defined as −5,000 bp to +500 bp from TSS. **(B)** Comparison between the binding motifs of SP1 and YYN + YNN peaks.

To identify chromatin loci where mediator activity might be regulated by CARM1, we determined the chromatin-associated overlap of MED12, CARM1, and CARM1 activity (H3R17me2a) by ChIP-seq in MCF-7 cells. To determine the chromatin distribution of methylated MED12, we developed two methyl-specific MED12 antibodies (meMED12^a&b^), as mentioned above. In addition to methylated MED12, the antibodies recognized at least one other CARM1 substrate (Fig 3A), and are thus not ideal for ChIP experiments. The H3R17me2a antibody (Millipore) recognizes a number of different CARM1 substrates, including MED12 (Fig S1A), SRC-3 (Fig S1F), CA150 (Cheng et al, 2007), and SmB (Cheng et al, 2007). The H3R17me2a antibody is not totally pan because it does not recognize the methyl-motifs on KMT2D and SRC-2 (Fig 2B). Therefore, ChIP-seq with this antibody provides a genomic readout for most CARM1 activity, not just for the histone mark alone. From the ChIP-seq data, we detected 992 MED12 binding sites, 743 CARM1 binding sites, and 726 peaks enriched for CARM1 activity in proliferating MCF-7 cells, grown in phenol red–containing media. We observed a 33% (410 of a total of 1,234 binding sites) overlap of the CARM1, MED12, and H3R17me2a profiles (Fig 5A). Analysis of the overlapping peaks identified a number of estrogen-regulated genes. We then compared our ChIP-seq data with the binding profiles of ERα and various histone modifications associated with “active” enhancers (H3K4me1 and H3K27ac), promoter (H3K4me3), and repressed (H3K27me3) regions (Fig 5B). ChIP-seq peaks from all three experiments correlate highly with functional enhancers and moderately with active promoters, but show no overlap with the repressed regions. In addition, our ChIP-seq data also displayed good overlap between H3R17me2a, active enhancer marks, and the enhancer-bound protein, MED12, which agrees with the ChIP-on-chip studies performed by Myles Brown's group that reported CARM1 activity predominantly at enhancer regions in MCF-7 cells (Lupien et al, 2009). Importantly, 60% of the peaks co-occupied by CARM1, MED12, and H3R17me2a (category YYY) overlap with ERα peaks, indicating that these mediator-bound regions targeted by CARM1 are ERα-specific enhancers. The H3K27ac and H3K4me1 marks decorate the edges of active enhancers, creating a “trough” where the transcription factors and coregulators are enriched. It is in this trough that we see CARM1, MED12, CARM1 activity (H3R17me2a) and ERα signals (Fig 5C). In addition, motif analysis of CARM1, MED12, and CARM1 activity peaks revealed a consensus sequence that is almost identical to the canonical ERα binding motif (Fig 5D). It is noteworthy that a subset of peaks, which are weak in ERα and H3R17me2a signals, are associated with active promoters (i.e., categories YYN and YNN overlap well with the H3K4me3 signal) (Figs 5B and S3A). We speculate that these peaks may constitute a distinct class of CARM1-regulated genes that do not associate with ERα. Indeed, motif analysis under this subset of H3K4me3 peaks identified a binding motif for Sp1 (Fig S3B), suggesting that this basal transcription factor may also make use of CARM1's coactivator activity.

**Figure 6.**
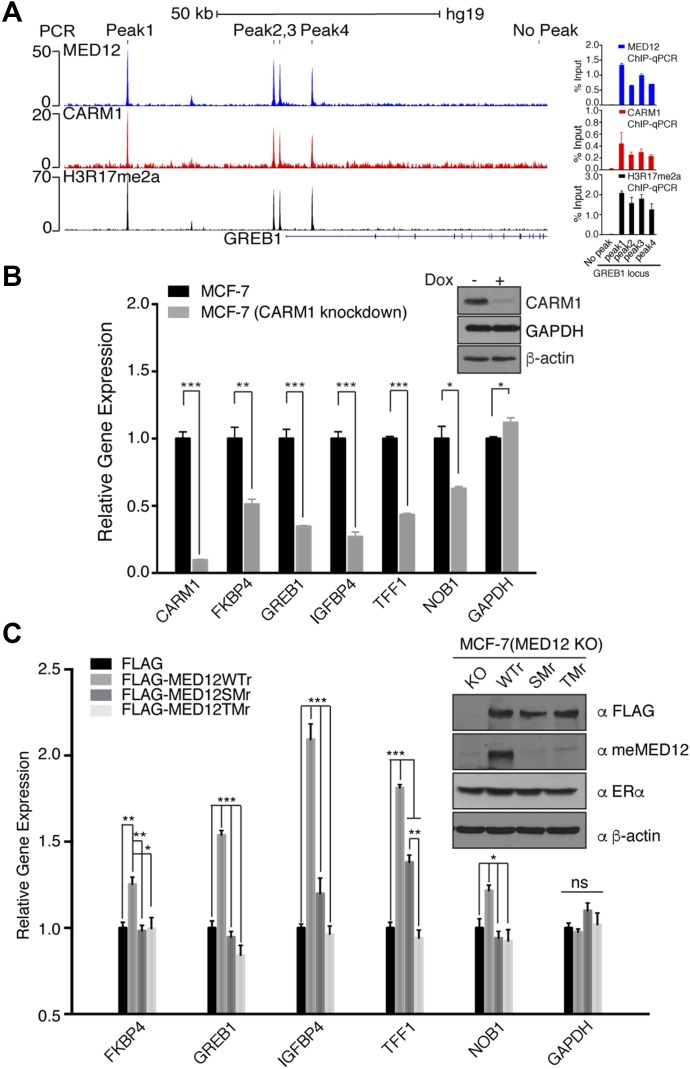
Regulation of ChIP-seq target genes by MED12 methylation. **(A)** ChIP-seq peaks demonstrating the enrichment of MED12, CARM1, and H3R17me2a signals at the *GREB1* gene locus (left panel). ChIP and quantitative PCR analysis of the association of MED12, CARM1, and H3R17me2a with *GREB1* gene (right panel). **(B)** MCF-7-Tet-on-shCARM1 cells were untreated or treated with doxycycline (1 μg/ml) for 8 d. Total RNA was extracted and RT-PCR was performed using primers specific for the genes shown. GAPDH acts as a negative control. Target gene expression was normalized to β-actin. Error bars represent SD based on three independent experiments. *P < 0.05, **P < 0.01, and ***P < 0.001 (*t* test). **(C)** A rescue experiment was performed whereby WT, single (R1899K), or triple (R1862K/R1899K/R1912K) mutant FLAG-MED12 constructs were reintroduced into MED12 KO MCF-7 cells generated by CRISPR-Cas9 gene editing. These constructs (denoted by WTr, SMr, and TMr) contain five synonymous mutations in the guide sequence to prevent Cas9 cleavage. MED12 KO cells were cultured in phenol red–free DMEM supplemented with 10% charcoal dextran-stripped FBS for 3 d before transfection, and then treated with E2 (50 nM) for 24 h. Total RNA was extracted and RT–qPCR was performed using primers specific for the genes shown. Target gene expression was normalized to β-actin. Inset figures show similar expression levels of the WT and mutant proteins. Error bars represent SD based on three independent experiments. *P < 0.05, **P < 0.01, and ***P < 0.001 (*t* test).

**Figure S4.**
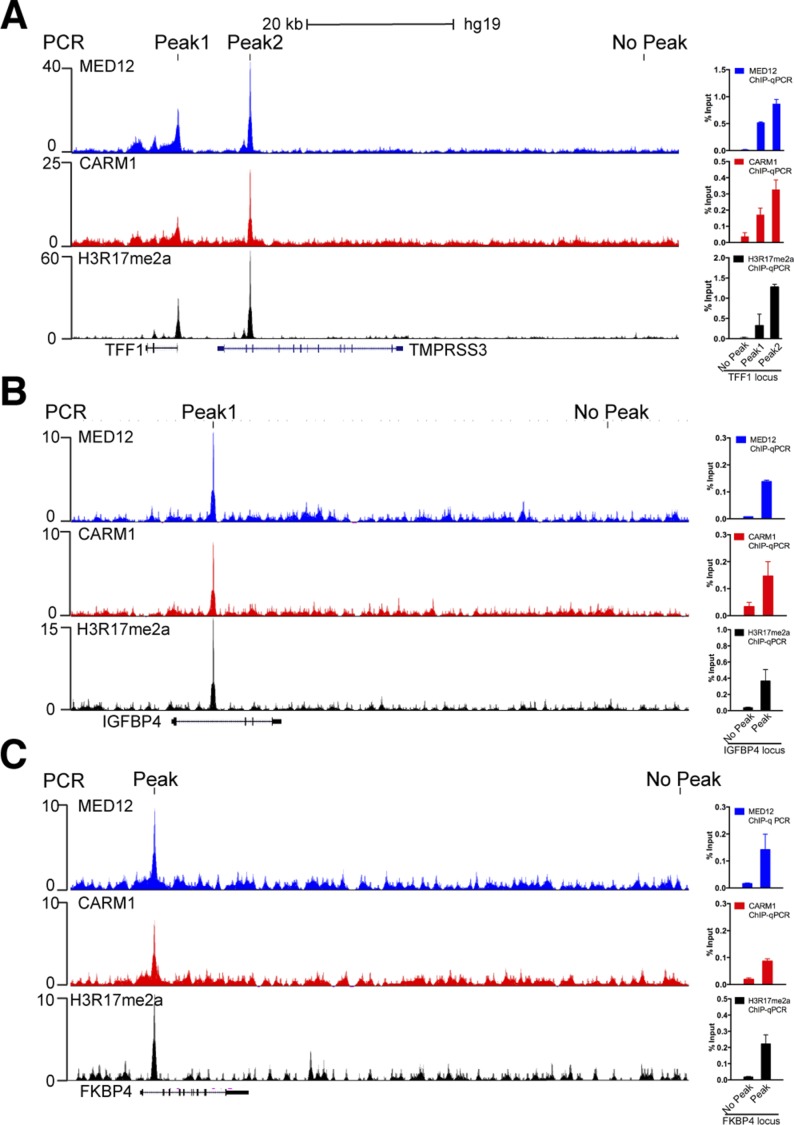
Confirmation of ChIP-seq peaks for the target genes. ChIP-seq peaks and qPCR analysis demonstrating the enrichment of MED12, CARM1, and H3R17me2a signals at *TFF1* (A), *IGFBP4* (B), and *FKBP4* (C) gene loci in MCF-7 cells.

**Figure S5.**
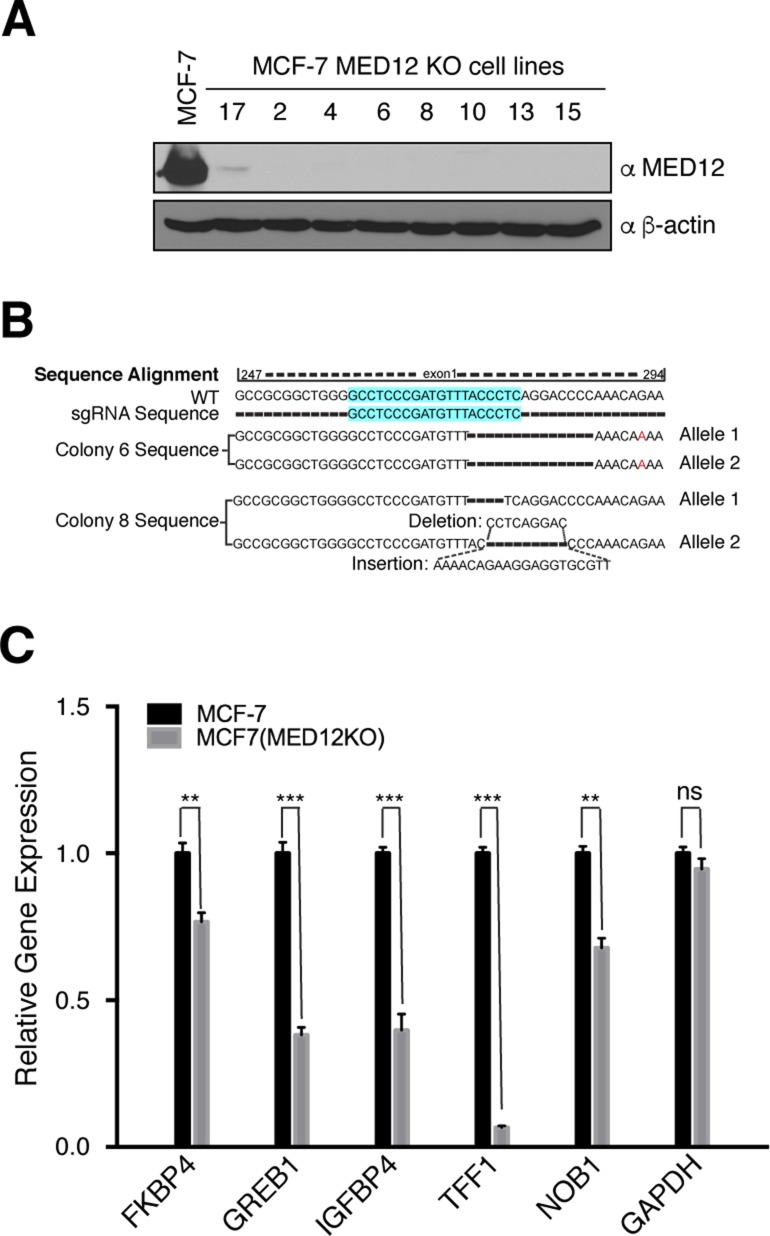
Generation of MED12 KO MCF-7 cells using CRISPR/Cas9. **(A)** Western blot analysis of the MED12 KO MCF-7 clones (−/−) together with parental cells (WT). β-actin serves as a loading control. **(B)** Sequencing analyses of two MED12 KO clones. Target sequence has been highlighted in blue. Representative indel sequences are shown. **(C)** The expression of the indicated ERα target genes significantly decreased in MED12 KO MCF-7 cells compared to their parental counterparts. Total RNA was extracted and RT–qPCR was performed using primers specific for the genes shown. GAPDH acts as a negative control. Target gene expression was normalized to β-actin. Error bars represent SD based on three independent experiments. *P < 0.05, **P < 0.01, and ***P < 0.001 (*t* test).

To highlight the degree of co-occupancy of CARM1, MED12, and CARM1 activity at EREs, we focused on the well-characterized ERα target gene, *GREB1*, which has four well-defined EREs (Carroll et al, 2006) (Fig 6A). Similar tight overlap of these three ChIP-seq profiles is also observed at the *TFF1*, *IGFBP4*, and *FKBP4* loci (Fig S4A–C). Next, five ERα target genes that displayed strong overlap of all three ChIP-seq profiles (*GREB1*, *TFF1*, *IGFBP4*, *FKBP4*, and *NOB1*) were tested for their dependency on CARM1 for optimal E2-induced expression. To do this, a Tet-inducible CARM1 shRNA-knockdown MCF-7 cell line was used, and CARM1 knockdown significantly reduced the expression of all five tested genes (Fig 6B). To establish the importance of the MED12 methylation sites in this process, we generated a CRISPR-mediated MED12 KO MCF7 cell line (Fig S5A–C), and rescued this cell line with WT and mutant forms of MED12—either a single mutant (SM) at R1899, or a triple mutant (TM) at R1862, 1899, and R1912. Although the reexpression of WT MED12 was able to rescue the expression of this panel of ER-regulated genes, neither of the mutant MED12 vectors were capable (Fig 6C), thus highlighting the importance of the R1899 methylation site on MED12.

### MED12 methylation by CARM1 regulates its ability to bind ncRNA-a

**Figure 7.**
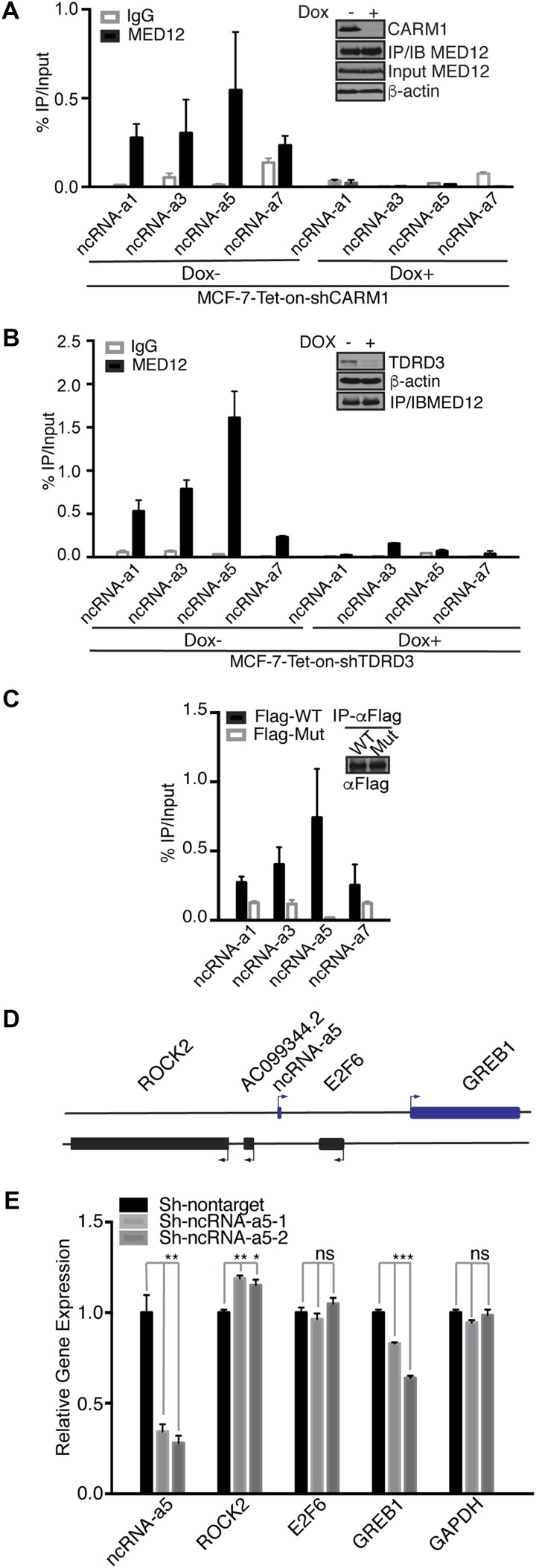
MED12 association with ncRNA-a is disrupted by loss of CARM1 and TDRD3. **(A)** MCF-7-Tet-on-shCARM1 cells were untreated or treated with doxycycline (1 μg/ml) for 8 d to knockdown CARM1 (see inset). Lysates were subjected to UV-RIP using IgG or MED12 antibodies and analyzed by RT–qPCR with primers specific for the indicated ncRNA-a. Error bars represent SD based on replicates (n = 3). **(B)** MCF-7-Tet-on-shTDRD3 cells were induced with doxycycline (1 μg/ml) for 8 d to knockdown TDRD3 (see inset). The cells were subjected to UV crosslinking followed by RNA IP (UV-RIP) using IgG or MED12 antibody and analyzed by RT–qPCR with primers specific for the indicated ncRNA-a. Error bars represent SD based on replicates (n = 3). **(C)** HEK293T cells transiently expressing FLAG-MED12 (WT) or FLAG-MED12R1899K (Mut) were UV crosslinked, lysed, and incubated with FLAG antibody. The immunoprecipitated RNAs were then analyzed by RT–qPCR to assess ncRNA-a levels. Inset shows similar immunoprecipitated levels of WT and Mut proteins. **(D)** ncRNA-a5 is located near *GREB1* on human chromosome 2p25.1, along with *E2F6* and *ROCK2* genes. **(E)** Knockdown of ncRNA-a5 results in reduced expression of GREB1, as gauged by RT–qPCR. This experiment was performed in MCF-7 cells induced with E2 for 4 h prior to RNA isolation. RT–qPCR was performed using primers specific for the genes shown. Target gene expression was normalized to β-actin. Error bars represent SD based on three independent experiments. *P < 0.05, **P < 0.01, and ***P < 0.001 (*t* test).

**Figure S6.**
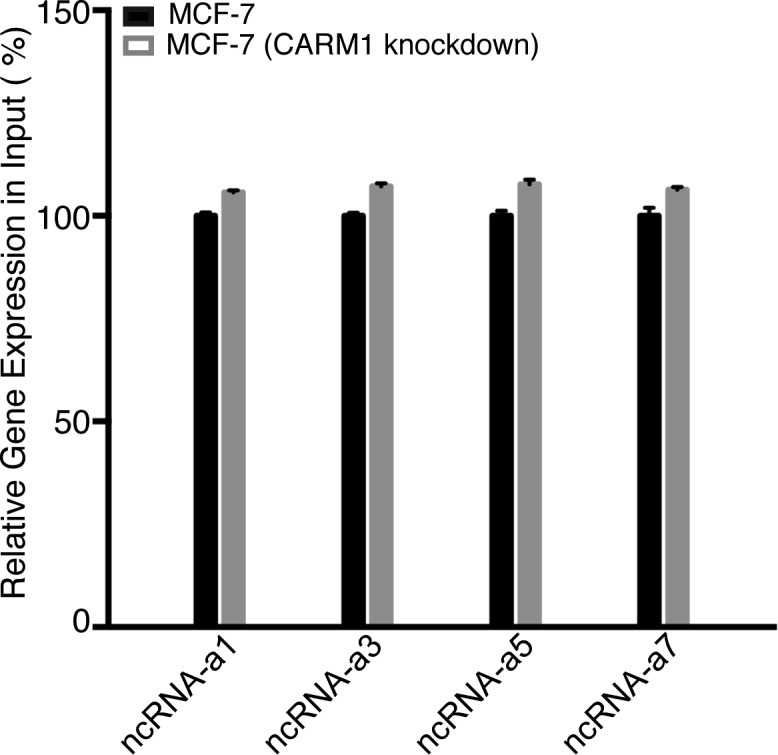
CARM1 knockdown does not alter the levels of ncRNA-a. RT–qPCR analysis demonstrating similar ncRNA-a levels in the input samples of CARM1 WT and knockdown MCF-7 cells.

There is emerging evidence that the mediator complex interacts directly with RNA at enhancer elements (Kim & Shiekhattar, 2015). In particular, two studies have recently shown that the appearance of bi-directional enhancer RNAs (eRNAs) correlate with the recruitment of the mediator complex (Hsieh et al, 2014; Step et al, 2014), and siRNA knockdown of eRNA production results in reduced mediator recruitment (Hsieh et al, 2014). In addition, a direct interaction between MED12 and a class of long noncoding RNAs called activating ncRNAs (ncRNA-a) has been identified (Lai et al, 2013). Activating ncRNAs function by regulating their neighboring genes using a cis-mediated mechanism (Orom et al, 2010; Wang et al, 2011). Recently, Dlx1as has also been identified as an ncRNA-a that binds MED12 and controls the expression of members of the nearby HoxD gene cluster (Papadopoulou et al, 2016). To investigate whether CARM1 may regulate the MED12/ncRNA-a interactions, we performed an RNA IP (RIP) experiment with MED12 antibodies, followed by RT–qPCR. We found that a set of ncRNAs interact with MED12 in a CARM1-dependent manner (Fig 7A). Importantly, the loss of ncRNA-a interaction with MED12 in CARM1 knockdown cells is not due to decreased expression of these ncRNAs (Fig S6). Furthermore, this interaction was dependent on TDRD3 (Fig 7B), the methyl-dependent MED12 interacting protein we identified (Fig 4). Also, the MED12-R1899K mutant interacted less efficiently with the tested ncRNA-a set (Fig 7C). Interestingly, TDRD3 recruits TOP3B to not only target R-loops (Yang et al, 2014), but also unwinds RNA (Stoll et al, 2013), which may be important here. Also, TDRD3 binds directly to single-stranded RNA (Siaw et al, 2016). To test the hypothesis that CARM1 methylation of MED12 could regulate the transcription of a gene adjacent to an ncRNA-a locus, we focused on ncRNA-a5, which lies close to the well-documented CARM1-regulated ER target gene, *GREB1* (Rae et al, 2005) (Fig 7D). Knockdown of ncRNA-a5, using two independent shRNAs, selectively reduces the expression of *GREB1* (Fig 7E).

## Discussion

### CARM1 methylates an ill-defined, but distinct, proline-rich motif

Most PRMTs methylate GAR domains (Thandapani et al, 2013); CARM1 does not. When the methylation site in CARM1 substrates is aligned (Lee & Bedford, 2002; Cheng et al, 2007; Shishkova et al, 2017), there is clearly no obvious motif, except for the propensity for proline residues in the vicinity of the CARM1 methylation site. But even the proline residues are not at a fixed position from the methylated arginine, suggesting that all that is needed is a stiff bend, on either side (or both sides) of the methylation site. Indeed, using oriented peptide arrays, a similar proline-rich methylation motif was identified for CARM1, in a totally unbiased manner (Gayatri et al, 2016). Thus, even after the identification of a number of different CARM1 substrates, the methylation motif for this enzyme still remains rather nebulous and difficult to predict based on the primary sequence. Interestingly, although it is difficult to predict a CARM1 substrate, antibodies developed to one substrate often cross-react with other substrates, so antibodies are able to identify some structural similarity between CARM1 methylation motifs. This “semi-pan” nature of CARM1 substrate antibodies was first realized using the αH3R17me2a antibody, which we have shown recognizes SRC3 (Naeem et al, 2007) and CA150 (Cheng et al, 2007), and also recognizes GPS2, SLM2, PABP1, SF3B4, SRC1, and MED12 (Fig 2B).

Given the transient nature of CARM1-substrate interaction, it has been challenging to obtain cocrystal structures of these complexes. However, structural studies using three diverse substrate sequences provided insight into the flexibility of the CARM1 enzyme (Boriack-Sjodin et al, 2016), which were further supported using novel transition state mimics of two independent CARM1 substrate motifs (van Haren et al, 2017). These two studies revealed that the binding interactions of the peptides with CARM1 were permissive of flanking proline residues on either side of the substrate arginine. Furthermore, the conformational constraints bestowed on a peptide motif by proline residues, kinks the arginine residue into the active site.

### CARM1 primarily associates with enhancers, but is also found at promoters

**Figure S7.**
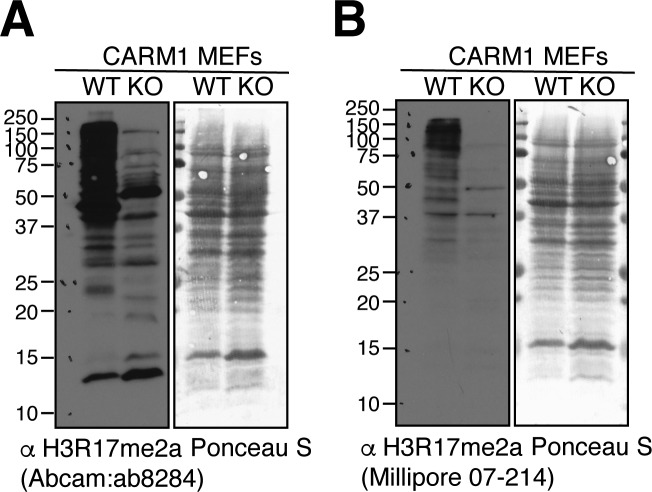
Characterization of H3R17me2a antibodies. Whole cell extracts from CARM1 WT (+/+) and KO (−/−) MEFs were subjected to Western analysis with αH3R17me2a (ab8284; Abcam, left panel) and αH3R17me2a (07-214; Millipore, right panel) antibodies. Ponceau S staining shows roughly equal loading.

By exploiting the pan nature of the H3R17me2a antibody (Millipore), CARM1 activity was first mapped in a global fashion by Myles Brown's group, using a ChIP-on-chip approach (Lupien et al, 2009). They found that the majority (70%) of ERα binding sites are associated with CARM1 activity. Most of the CARM1 activity mapped to intergenic and intronic regions, with less than 3% of this activity associated with proximal promoter regions. Our expanded H3R17me2a ChIP-seq data support these findings. Interestingly, recent H3R17me2a ChIP-seq experiments using an antibody from Abcam, mapped CARM1 activity to promoter regions in MEFs (Shin et al, 2016). These disparities could be due to the different cell types (MCF7 versus MEF) or antibodies used in the different experiments. Importantly, the two independently sourced H3R17me2a antibodies (Millipore and Abcam) both recognize many CARM1 substrates when tested by Western blot on total cell lysates (Fig S7), so clearly, neither antibody can be used to identify the chromatin localization of the H3R17me2a histone mark, without recognizing other CARM1 substrates.

The association of CARM1 itself with chromatin has been investigated using a reChIP-on-chip approach (Coughlan et al, 2013). In this study from Joe Torchia's group, a promoter array was used for the profiling, so they were not able to investigate enhancer enrichment of CARM1. However, the SRC3/CARM1 protein complex associated not only with promoter proximal EREs, but also with Sp1 and C/EBPα binding motifs (Coughlan et al, 2013). Importantly, the Sp1 motif is enriched in the YYN and YNN clusters that are associated with CARM1 activity at promoters that are also marked with H3K4me3 signal (Figs 5B and S3). We did not observe enrichment for the C/EBPα motif in our ChIP-seq experiment. The reChIP-on-chip experiment focused on identifying promoters that recruited CARM1 through its association with SRC3 (Coughlan et al, 2013). This is likely just a subset of promoters that are engaged by CARM1 because it can be recruited by other SRCs and also directly by other transcription factors themselves. Indeed, CARM1 interacts directly with ERα (without the help of SRCs) in response to cAMP signaling (Carascossa et al, 2010), and also with c-Fos (Fauquier et al, 2008) and C/EBPβ (Kowenz-Leutz et al, 2010). CARM1 not only binds, but also methylates C/EBPβ and inhibits the association of this transcription factor with the mediator complex (Kowenz-Leutz et al, 2010). NF-κB is another transcription factor that directly interacts with CARM1 (Covic et al, 2005), and it was later reported that CARM1 is present at the promoter/enhancer looping joint of a NF-kB–regulated gene (*MCP-1*) (Teferedegne et al, 2006). In this setting, CARM1 was not required for looping, but was required for efficient expression of *MCP-1*. CARM1 also binds directly to the Notch intracellular domain, and can be detected at the notch intracellular domain–bound enhancer sites of a number of notch target genes (Hein et al, 2015). Thus, CARM1 is not a dedicated ERα coactivator, but is also recruited by other transcription factors to both proximal promoters and enhancer elements.

CARM1 is not only recruited to chromatin by transcription factors, but also by ATP-dependent remodeling complexes, including the SWI/SNF complex (Xu et al, 2004; Wang et al, 2014), and directly interacts with the Mi2α and Mi2β, components of the NuRD complex (Streubel et al, 2013). Although NuRD is generally considered a transcriptional repressor complex, there is emerging evidence that it can also positively regulate gene expression, particularly in light of recent genomic localization studies that show its enrichment at active promoters and enhancers (Shimbo et al, 2013).

At both enhancer elements and at some proximal promoters, CARM1 methylates a host of different proteins that are detected with the H3R17me2a antibody. However, it is clear that the H3R17me2a Millipore antibody does not recognize all CARM1 substrates (Fig 2B—KMT2D and SRC2). This is supported by our ChIP-seq data showing two categories (YYN and YNN) that display strong CARM1 recruitment, but very little H3R17me2a antibody ChIP signals (Fig 5B). It is likely that CARM1 activity at these proximal promoter regions will be detected using a different CARM1 substrate antibody. These data support the idea of distinct methylarginine “fingerprints” on transcriptional coactivators at different gene promoters and enhancers, which was first proposed by the Gronemeyer group (Ceschin et al, 2011). In that study, they showed that three methyl-specific antibodies, raised against different CARM1 methylation sites on the CBP, displayed dissimilar localization signatures at different EREs. In a similar way, it is possible that MED12, which has at least three prominent CARM1 methylation sites (R1862, R1899, and R1912), will also be modified in different combinations to exert different MED12 functions.

### How does CARM1 methylation of MED12 regulate its function?

MED12 has cytoplasmic and nuclear functions. In the cytoplasm, it regulates the TGF-βR2 pathway (Huang et al, 2012). Methylation of MED12 by CARM1 at R1862 and R1912 regulates its cytoplasmic functions (Wang et al, 2015). We demonstrate here that methylation of MED12 at R1899 regulates a subset of its nuclear functions, and provides an additional level of gene expression regulation governed by CARM1. Although the mediator complex broadly regulates transcription, the MED12 subunit is only partially methylated in cells (Fig 3D), suggesting that the mediator complex targeted by CARM1 may regulate a specific set of transcriptional programs, as opposed to all RNA Pol II-dependent genes. From ChIP-seq experiments, we defined this specific set of genes to be ERα-dependent and confirmed, by gene expression studies, that MED12 methylation enhanced the transcriptional activation of these genes (Fig 6B and C).

**Figure 8.**
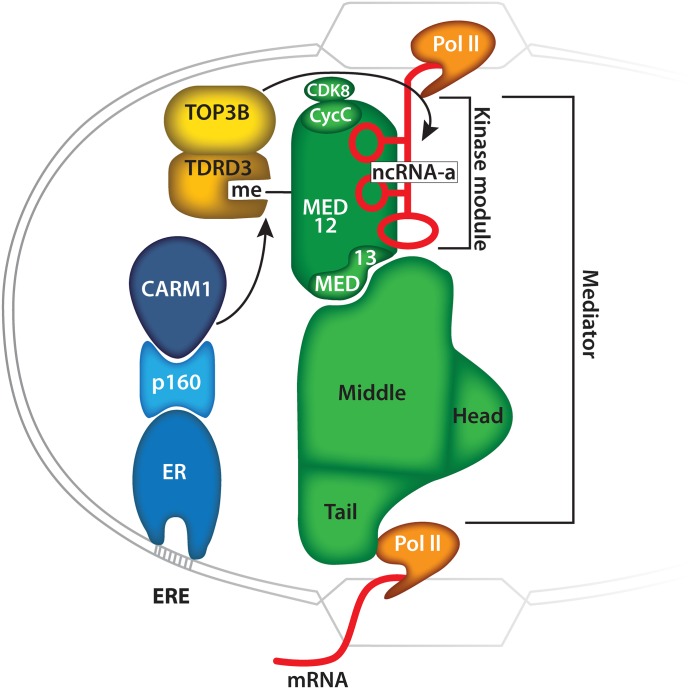
A model representing the regulation of MED12/ncRNA-a interactions by CARM1. The sizes of the different proteins and protein complexes are not drawn to scale.

Methylation of MED12 signals the recruitment of TDRD3, which may promote transcriptional activation because of TDRD3's coactivator activity (Yang et al, 2010). TDRD3 is in a tightly bound complex with the topoisomerase, TOP3B (Stoll et al, 2013; Yang et al, 2014). It has been shown that the TDRD3/TOP3B complex is recruited to active chromatin through the ability of the TDRD3 Tudor domain to interact with the H3R17me2a and H4R3me2a marks (Yang et al, 2014), and the C-terminal domain of RNA Polymerase II (Sims et al, 2011). Here, we report a third mark that is “read” by TDRD3: MED12R1899me2a. The recruitment of the TDRD3/TOP3B complex to this motif could help resolve R-loops at sites of active transcription (Yang et al, 2014), or it could act on ncRNA molecules that are associated with the mediator complex. Indeed, TOP3B was recently shown to possess both DNA and RNA topoisomerase activities (Xu et al, 2013; Yang et al, 2014; Siaw et al, 2016). Importantly, the mediator has been shown to associate with at least two classes of noncoding RNAs: (1) activator RNAs that increase the transcription of neighboring genes (Lai et al, 2013) and (2) eRNAs that correlate with enhancer–promoter looping and gene activation (Hsieh et al, 2014). The recruitment of the TDRD3/TOP3B complex, and its dual topoisomerase activity, may be required for not only reducing R-loop formation in the wake of Pol II at active genes, but also for the “untangling” and “correct” structural presentation of these RNA scaffolds for efficient MED12 binding, at sites of enhancer–promoter looping (Fig 8).

## Materials and Methods

### Antibodies

CARM1 substrate motif antibodies were raised against an antigen mixture of six different CARM1 methylated motifs (Fig 1A), to specifically recognize endogenous proteins when asymmetrically dimethylated by CARM1. The meMED12 antibodies were raised against the peptide sequence TSVYR*QQQP of human MED12 protein (NP_005111). This work was performed in collaboration with Cell Signaling Technology (CST). mePABP1 antibody was raised against the peptide sequence CGAIR*PAAPR*PPFS of human PABP1 protein (NP_002559) (Cheng & Bedford, 2011). R* denotes asymmetrically dimethylated arginine residue. The MED4, MED30, and CDK8 antibodies were a gift from Thomas Boyer (University of Texas Health Science Center at San Antonio). The CARM1 antibody, used for ChIP-seq, was a gift from Stéphane Richard (McGill University). The following antibodies were obtained commercially: H3R17me2a (07-214; Millipore and ab8284; Abcam), MED12 (A300-774A; Bethyl), SRC-1 (2191; CST), SRC-3/AIB1 (611105; BD Transduction), CARM1 (A300-420A; Bethyl), PRMT1 (A300-722A; Bethyl), PRMT6 (A300-929A; Bethyl), CDK8 (SC-1521; Santa Cruz), and FLAG (F7425 [rabbit IgG] and F3165 [mouse IgG]; Sigma-Aldrich).

### Plasmids and peptides

GST-PABP1 (Lee & Bedford, 2002), GST-CARM1 (Frankel et al, 2002), FLAG-CA150 (Cheng et al, 2007), GST-Tudor (TDRD3) (Yang et al, 2010), and GST-Tudor (SMN) (Kim et al, 2006) have been described previously. The other GST-Tudor constructs used in the pull-down experiments were generated by cloning the Tudor domains of SPF30, SPIN1, SND1, and TDRKH, separately, into a pGEX-6p-1 vector (Biomatik). Recombinant H3 protein used in the in vitro methylation assay was purchased from New England Biolabs. The siRNA-resistant p3XFLAG-MED12r plasmid was a gift from Thomas Boyer (University of Texas Health Science Center at San Antonio). The KMT2D^a^ fragment (3,619–4,285 aa) was amplified from human cDNA using gene-specific primers and subcloned into a p3XFLAG-CMV-7.1 vector. FLAG-GPS2 was a gift from Darryl Zeldin (NIEHS). SLM2 cDNA was a gift from Stéphane Richard (McGill University). FLAG-PABP1 and FLAG-SF3B4 were generated by cloning the cDNAs from GST-PABP1 and His-SF3B4 (Lee & Bedford, 2002; Cheng et al, 2007). SRC-1, SRC-2, and SRC-3 constructs were kindly provided by Bert O'Malley (Baylor College of Medicine). FLAG-MED12-R1899K and FLAG-KMT2D^a^-R3727K mutants were generated using a quick-change site-directed mutagenesis kit (Agilent). Biotinylated peptides encompassing residues 1,891–1,907 of the MED12 protein with unmodified, or asymmetrically dimethylated, Arg1899 were purchased from CPC Scientific.

### Cell lines

The CARM1 WT and KO MEFs (Yadav et al, 2003), the tamoxifen-inducible PRMT1^fl/−^ ER-Cre MEFs (Yu et al, 2009), and the tetracycline-inducible T-REx-CARM1-293 cell line (Cheng et al, 2007) have been described previously. The MCF-7-Tet-on-shCARM1 cell line was a gift from Wei Xu (University of Wisconsin). MCF-7, HeLa, and HEK293T cell lines were obtained from ATCC. To generate MED12 KO MCF-7 cell lines, a 20 bp guide sequence (GCCTCCCGATGTTTACCCTC) targeting the first exon of MED12 was selected using the online software tool, ZiFiT Targeter. Two complementary oligos (5′-CACCGCCTCCCGATGTTTACCCTC-3′ and 5′-AAACGAGGGTAAACATCGGGAGGC-3′) containing the guide sequence were cloned into the LentiCRISPR vector, which also expresses Cas9 and a puromycin selectable marker for targeted KO. To generate ncRNA-a5 stable knockdown MCF-7 cell lines, SMARTvector lentiviral vector encoding nontargeting control-shRNA (VSC11707; Dharmacon) or SMARTvector lentiviral vectors encoding each of two distinct ncRNA-a5 (V3SH11246-245210427 and V3SH11246-245589794; Dharmacon) were used. Infected MCF-7 cells were selected using puromycin (2 μg/ml) to develop the lines. For MED12 KO, single cell clones were validated by Western blotting and confirmed by Sanger sequencing (using 5′-GAGGGATCCCTCGGCTTCCCTCGGTAGTTTC-3′ and 5′-GAGGTCGACCCCTATTCATACCTTGGAACCC-3′ primers). All cell lines were maintained in Dulbecco modified Eagle medium containing 10% fetal bovine serum.

### MS

These experiments were performed at CST using an approach developed by John Rush for the identification of tyrosine phosphorylation sites (Rush et al, 2005). Briefly, proteins were extracted from the WT MEF cell line and digested with trypsin. The resulting complex peptide mixture was partitioned into three fractions by reversed-phase solid-phase extraction, and each fraction was treated with one of the four CARM1 substrate antibodies immobilized on agarose beads. After washing, peptides were eluted and analyzed by nanoflow LC-MS/MS. The resulting spectra were assigned to peptide sequences using the program Sequest. Lists of credible methylpeptide sequence assignments were generated.

### In vitro methylation assay

The in vitro methylation reactions contained 1 μg substrate (PABP1, H3, or MED12 peptides), 1 μg recombinant GST-CARM1, and 1 μl S-adenosyl-L-[methyl-^3^H] methionine (81.7 Ci/mmol from a 6.7 μM stock solution, NET155001MC; PerkinElmer) in a final volume of 30 μl PBS. The reactions were incubated at 30°C for 1.5 h, and boiled in protein loading buffer for 5 min. The samples were resolved by SDS–PAGE, transferred onto polyvinylidene difluoride membranes, treated with En^3^hance (6NE970C; PerkinElmer), and exposed to film for 1–3 d at −80°C.

### Peptide pull-down assay

Biotinylated MED12 peptides (20 μg) were immobilized on 20 μl streptavidin agarose beads (16–126; Millipore) in 500 μl of mild lysis buffer (50 mM Tris–HCl pH 7.5, 150 mM NaCl, 0.1% Nonidet P-40, 5 mM EDTA, 5 mM EGTA, and 15 mM MgCl_2_) at 4°C for 2 h. The beads were washed three times with mild lysis buffer and incubated with 4 μg of GST-Tudor protein in 500 μl mild lysis buffer at 4°C for 2 h. After three washes with mild lysis buffer, the beads were boiled in protein loading buffer and subjected to Western blot analysis using αGST antibody.

### IP

HEK293T cells (90% confluent) were transiently transfected with expression vectors encoding FLAG-tagged putative CARM1 substrates using Lipofectamine 2000 (Invitrogen). Cells (10 cm plate) were harvested after 24 h, washed with ice-cold PBS and lysed in 1 ml of radioimmunoprecipitation assay buffer (25 mM Tris–HCl pH 7.6, 150 mM NaCl, 1% Nonidet P-40, 1% sodium deoxycholate, 0.1% sodium dodecyl sulfate, and 2 mM EDTA) with protease inhibitor cocktail (Roche). The lysates were incubated with 40 μl of anti-FLAG M2 affinity gel (A2220; Sigma-Aldrich) for 1 h. The beads were washed three times with radioimmunoprecipitation assay buffer, eluted in protein loading buffer and analyzed by Western blotting. For the co-IP assays, cells (10 cm plate) were lysed in 1 ml of mild lysis buffer and incubated with the specified antibodies. Immunoprecipitates were pulled down using protein A/G ultralink resin (53132; Thermo Fisher Scientific) and the eluted proteins were analyzed by Western blotting. For the TDRD3-MED12 co-IP experiment, MCF7-tet-on-shCARM1 cells (Yang et al, 2010) were treated with 1 μg/ml of doxycycline for 6 d to knockdown endogenous CARM1 expression. Untreated parental cells were used as controls. Cells were lysed in buffer A (10 mM Hepes, 1.5 mM MgCl_2_, 10 mM KCl, 0.5 mM DTT, 0.05% NP40 pH 7.9) supplemented with cocktails of protease inhibitor and phosphatase inhibitor (Pierce) for 10 min on ice. After centrifugation at 4°C at 845 *g* for 10 min, the supernatant was removed and the pellet was resuspended in 387 μl of buffer B (5 mM Hepes, 1.5 mM MgCl_2_, 0.2 mM EDTA, 0.5 mM DTT, 26% glycerol [vol/vol] pH 7.9) supplemented with 13 μl of 4.6M NaCl to give 150 mM NaCl. After lysing on ice for 10 min, brief sonication was applied to dissolve the pellet. Cell lysates were kept on ice for an additional 30 min. After centrifugation at 24,000 *g* for 20 min at 4°C, the supernatant was collected as nuclear extract for IP using anti-TDRD3 antibody.

### ChIP and quantitative PCR

Chromatin was harvested from MCF-7 cells as described previously (Iberg et al, 2008) and ChIP was performed using CARM1, MED12, and H3R17me2a antibodies. Using 2 μl of ChIP DNA as the template, qPCR was performed on the ABI 7900 HT fast real-time PCR system with primer sets against the specified genes (refer to Table S1). The data were analyzed using the Sequence Detection System software (ABI). The experimental cycle threshold (Ct) was calibrated against the input product.

Table S1 ChIP-qPCR primers.

### ChIP-seq analysis

#### Mapping of reads

Sequenced DNA reads were mapped to the human genome hg19 using bowtie (version 0.12.8) (Langmead et al, 2009) and only the reads that were mapped to a unique position were retained. 31–63 million reads were generated per sample. 80–97% reads were mapped to the human genome, with 59–71% uniquely mapped. To avoid PCR bias, for multiple reads that were mapped to the same genomic position, only one copy was retained for further analysis. In the final, 22–41 million reads were used in peak calling and downstream analyses.

#### Peak calling and gene annotation

The original peak calling for CARM1/MED12/H3R17me2a was performed by MACS (version 1.4.2) (Zhang et al, 2008) using total input DNA as the negative control. The window size was set as 300 bp and the *P*-value cutoff was 1 × 10^−6^. The peaks overlapping DAC blacklisted regions and Duke excluded regions downloaded from UCSC genome browser were removed. Then, the peaks from the three factors were merged (allowing at least 1 bp overlap) to form a highly confident superset of peaks. For each peak from the superset, if it overlapped the peaks of one of the factors called at *P*-value 1× 10^−4^, it was marked as occupied by the corresponding factor. Each peak in the superset was assigned to the gene that has the closest transcription start site (TSS) to it. Then, the peak was classified by its location to the gene: upstream (−50 k to −5 k from TSS), promoter (−5 k to +0.5 k from TSS), exon, intron, the transcription end site (TES) (−0.5 k to +5 k from TES), and downstream (+5 k to +50 k from TES). The gene list used to annotate the peaks is GENCODE release 19 (Harrow et al, 2012).

#### Landscape of ChIP-seq signal

Each read was extended by 150 bp to its 3′ end. The number of reads on each genomic position was rescaled to normalize the total number of mapped reads to 10 million and averaged over every 10 bp window. The normalized values were displayed in the UCSC genome browser.

#### Heatmap and average profile of ChIP-seq signal around peak center

10 kbp upstream and 10 kbp downstream from the center of each peak were subdivided into 250 bp bins. For each ChIP-seq sample, the reads per million reads per kilobase values for each bin were calculated and normalized through *z*-score transformation to minimize the potential batch effect. The values were then averaged over all peaks to generate the average profile or plotted in heatmap by R function heatmap.2.

#### Motif analysis

Motif analysis including de novo motif searching by Multiple Em for Motif Elicitation (MEME), identifying centrally enriched motifs by CentriMo, and matching of identified motifs to known motifs by Tomtom were all performed by the program MEME-ChIP (Machanick & Bailey, 2011) from MEME Suite (version 4.9.0). The sequences of −500 bp to +500 bp from the summit of peaks were taken as input. Motif Alignment & Search Tool from MEME suite was used to identify the existence of motifs in peaks (the *P*-value cutoff was set at 1× 10^−4^).

#### Published data sources

H3K4me3, H3K27me3, and H3K27ac ChIP-seq data were downloaded from UCSC genome browser “Histone Modifications by ChIP-seq from ENCODE/Stanford/Yale/UCS/Harvard” track (http://genome.ucsc.edu/cgi-bin/hgFileUi?db=hg19&g=wgEncodeSydhHistone). H3K4me1 ChIP-seq data were downloaded from Gene Expression Omnibus (GEO) dataset GSE57498. ERα ChIP-seq data were downloaded from GEO dataset GSE60270.

### Quantitative reverse transcription PCR (RT–qPCR)

MCF-7-Tet-on-shCARM1 cells were untreated or treated with doxycycline (1 μg/ml) for 5 d. The cells were then gently washed with PBS and transferred to phenol red–free DMEM supplemented with 10% charcoal dextran-stripped FBS. The cells were maintained in this media with or without doxycycline for 3 d and then treated with 50 nM E2 for 4 h. Total RNA was extracted using RNeasy Mini kit (74104; QIAGEN) and cDNA was synthesized using the Superscript III First-Strand Synthesis system (18080-051; Invitrogen). qPCR was then performed using primer sets against the specified genes (refer to Table S2). Data were analyzed using the Sequence Detection System software (ABI). The experimental Ct was calibrated against the β-actin control product, and the amount of sample product from Dox-treated cells relative to that of the control cells was determined using the DDCt method (onefold, 100%).

Table S2 RT–qPCR primers.

### UV cross-linking—RNA IP (UV-RIP)

UV cross-linking RIP was carried out as described previously (Hu et al, 2015) with modifications. Cells were washed with cold PBS and irradiated at 200 mJ/cm^2^ at 254 nm (Ultraviolet crosslinker, from Ultra-Violet Products, Limited Liability Company). Nuclei were collected using Nuclei Isolation Kit (nuc101; Sigma-Aldrich) and resuspended in 1 ml of RIP buffer (50 mM Tris–HCl pH 7.5, 150 mM NaCl, 1% NP40, 0.5% sodium deoxycholate, 1 mM PMSF, 400 U/ml RNase inhibitor, protease inhibitor cocktail). The nuclei were homogenized by sonication (10 cycles, 30 s “ON,” 30 s “OFF”) (diagenode; Bioruptor) and centrifuged at 13,000 rpm for 10 min at 4°C to remove the insoluble material. 50 µl of supernatant was saved as input. The rest of the supernatant was precleared by applying 15 μl of Dynabeads G (Invitrogen) with 20 μg/ml yeast tRNA for 1 h at 4°C. The precleared lysate was then incubated with 3 μg of IgG or MED12 antibodies overnight. The lysate was centrifuged at 1,587 *g* for 10 min at 4°C to remove the insoluble material and incubated with triple-washed Dynabeads G beads (20 μl) for 1 h at 4°C. The beads were then washed thrice (5 min each wash) using washing buffer I (50 mM Tris–HCl pH 7.5, 1 M NaCl; 1% NP40, 1% Sodium Deoxycholate, 2 mM ribonucleoside vanadyl complex and washing buffer II (50 mM Tris–HCl pH 7.5, 1 M NaCl, 1% NP40, 1% Sodium Deoxycholate, 2 mM VRC, 1 M urea). The immunoprecipitated complexes were eluted using 2 × 100 μl elution buffer (100 mM Tris–HCl at pH 7.0, 5 mM EDTA, 10 mM DTT, 1% SDS). Proteinase K (10 μg) was added into the 200 μl RNA sample and incubated for 30 min at 55°C. RNA was then extracted using RNeasy Mini Kit (74104; QIAGEN), digested with DNase I (QIAGEN), and used to synthesize cDNA using random hexamers (SuperScript III First-Strand Synthesis system 18080-051; Invitrogen) followed by qPCR analysis.

## Statistical Analysis

All the data are reported as sample mean ± SD. *t* test was performed to determine the *P*-value for RT–qPCR and RIP-qPCR experiments. The asterisks shows values of statistical significance, where * stands for *P*-value ranges between 0.01 and 0.05; ** stands for *P*-value ranges between 0.001 and 0.01, and ***stands for *P*-value less than 0.001.

## Supplementary Material

Reviewer comments
