# Supplementary material for: CARM1 methylates MED12 to regulate its RNA-binding ability
Source: Life Sci Alliance. 2018 Sep 19;1(5):e201800117. doi: 10.26508/lsa.201800117 (PMC6238599; doi:10.26508/lsa.201800117)
Supplement: Supplementary file 1 [file LSA-2018-00117_TableS1.docx]

**Supplementary table 1: ChIP-qPCR primers**

| **Gene Name** | **Forward Primer** | **Reverse Primer** |
| --- | --- | --- |
| *GREB1* No peak | TTTGCATGGACAGGCCTTGA | GCCCAAACCTTGGCACTTTC |
| *GREB1* peak1 | GAGAGGGTGGTGACACTTGG | CTGCAGCTGACAGAGGAGAC |
| *GREB1* peak2 | CTGGCTGCTTCCTGAGTGG | GCGTCAAGCAACTACACTCC |
| *GREB1* peak3 | TGAGCAAAAGCCACAAAGTAGT | TGCTGCGGCAATCAGAAGTA |
| *GREB1* peak4 | GGCTCCAGTCCAAGTACACA | AAATGCCACCGTTTCGTGT |
| *FKBP4* No peak | CTTCTCAGTAAGCCTGCGGT | GACTATCTGCCGAACCAGGG |
| *FKBP4* peak | CTCTGCTGTGGAGCCTGC | TCTCTGATTCTCCCTGGAACCT |
| *IGFBP4* No peak | TTTTGGGTCTGGGTGTGTGT | CAGCACCTGAGGAGTGCC |
| *IGFBP4* peak | AGGGTTGGGCAAGGAAAAGT | CATGCACTTGGGCACTCTGA |
| *TFF1* No peak | CCAGGGCCACCGAGAAC | CATTCTCGGGGTCAGCACC |
| *TFF1* peak 1 | ACATTTGCCTAAGGAGGCCC | ACCTCACCACATGTCGTCTC |
| *TFF1* peak 2 | ATTTCTTCTCCACGCCCTGT | GACAGCTGCCAGGTACGG |
