# Supplementary material for: CARM1 methylates MED12 to regulate its RNA-binding ability
Source: Life Sci Alliance. 2018 Sep 19;1(5):e201800117. doi: 10.26508/lsa.201800117 (PMC6238599; doi:10.26508/lsa.201800117)
Supplement: Supplementary file 2 [file LSA-2018-00117_TableS2.docx]

**Supplementary table 2: RT-qPCR primers**

| **Gene Name** | **Forward Primer** | **Reverse Primer** |
| --- | --- | --- |
| CARM1 | CCAGGGTGATGATGAAGGAC | GGGCATCGCCCTCTACA |
| FKBP4 | CTATCGTGGAGGTTGCACTG | CCATAAGGCAGATCCAGGTT |
| GREB1 | CTGTCCAGAGGGTGACATTG | CAGGGATGCTGCTTTAGTGA |
| IGFBP4 | AGGGGCTGAAGCTGTTGTT | CCTGCACACACTGATGCAC |
| NOB1 | TTGGAAGCAGAGTTTGTTGG | ATGTGCAGAGGTGTTTCTGG |
| TFF1 | TCCCTCCAGAAGAGGAGTGT | CAGAAGCGTGTCTGAGGTGT |
| ACTB | GTTGTCGACGACGAGCG | GCACAGAGCCTCGCCTT |
| GAPDH | AGCCACATCGCTCAGACAC | GCCCAATACGACCAAATCC |
| ncRNA-a1 | GCAAGCGGAGACTTGTCTTT | GGCTGGTCTTGAACTCCTGA |
| ncRNA-a3 | TTAAGCCCAAGGAATGGAGA | AGCGGTGTGGAATAAACTGG |
| ncRNA-a5 | ATGAGCCACTTCCAGCATC | CATCCCTTTCCTGGGGTAGT |
| ncRNA-a7 | CCGTTGGCTCCACAAACCT | CAGTGACAGTAGCAGGCATCCT |
| E2F6 | CTTCGCCATGAATCCTTCTC | TGGAGCCCATTCCTACATTC |
| ROCK2 | TGAAGCCTGACAACATGCTC | TACCATGCCTGTTTCATCCA |
