# Supplementary material for: CARM1 methylates MED12 to regulate its RNA-binding ability
Source: Life Sci Alliance. 2018 Sep 19;1(5):e201800117. doi: 10.26508/lsa.201800117 (PMC6238599; doi:10.26508/lsa.201800117)
Supplement: Reviewer comments [file LSA-2018-00117_review_history.pdf]

CARM1 Methylates MED12 to Regulate its RNA Binding Ability

Donghang Cheng, Vidyasiri Vemulapalli, Yue Lu, Jianjun Shen, Sayura Aoyagi, Christopher Frye, Yanzhong Yang, Charles Foulds, Fabio Stossi, Lindsey Trevino, Michael Mancini, Bert O'Malley, Cheryl Walker, Thomas Boyer, and Mark Bedford

DOI: 10.26508/lsa.201800117

Corresponding author(s): Mark Bedford, MD Anderson Cancer Center

Review Timeline:

Submission Date:	2018-06-25
Editorial Decision:	2018-07-20
Revision Received:	2018-08-15
Editorial Decision:	2018-09-03
Revision Received:	2018-09-06
Accepted:	2018-09-07

Scientific Editor: Andrea Leibfried

Transaction Report:

(Note: With the exception of the correction of typographical or spelling errors that could be a source of ambiguity, letters and reports are not edited. The original formatting of letters and referee reports may not be reflected in this compilation.)

July 20, 2018

Re: Life Science Alliance manuscript #LSA-2018-00117

Dr. Mark T. Bedford
MD Anderson Cancer Center
Epigenetics and Molecular Carcinogenesis
Science Park
Park road 1C
Smithville, Texas 78957

Dear Dr. Bedford,

Thank you for submitting your manuscript entitled "CARM1 Methylates MED12 to Regulate its RNA Binding Ability" to Life Science Alliance. The manuscript was assessed by expert reviewers, whose comments are appended to this letter. We invite you to submit a revision if you can address the reviewers' key concerns, as outlined here.

The reviewers provide constructive input and outline where further clarifications are needed (reviewers 1-3). A control to show loss of ncRNA binding for the R1899K mutant should be included, too (reviewer #1). The further reaching insight on the effects on the whole Mediator complex (reviewer #3) does not need to get experimentally provided for acceptance here.

To upload the revised version of your manuscript, please log in to your account:
https://lsa.msubmit.net/cgi-bin/main.plex
You will be guided to complete the submission of your revised manuscript and to fill in all necessary information.

While you are revising your manuscript, please also attend to the following editorial points to help expedite the publication of your manuscript. Please direct any editorial questions to the journal office.

A. THESE ITEMS ARE REQUIRED FOR REVISIONS

-- A letter addressing the reviewers' comments point by point.

-- An editable version of the final text (.DOC or .DOCX) is needed for copyediting (no PDFs).

-- High-resolution figure, supplementary figure and video files uploaded as individual files: See our detailed guidelines for preparing your production-ready images, http://life-science-alliance.org/authorguide

-- Summary blurb (enter in submission system): A short text summarizing in a single sentence the study (max. 200 characters including spaces). This text is used in conjunction with the titles of papers, hence should be informative and complementary to the title and running title. It should describe the context and significance of the findings for a general readership; it should be written in

the present tense and refer to the work in the third person. Author names should not be mentioned.

B. MANUSCRIPT ORGANIZATION AND FORMATTING:

Full guidelines are available on our Instructions for Authors page, http://life-science-alliance.org/authorguide

We encourage our authors to provide original source data, particularly uncropped/-processed electrophoretic blots and spreadsheets for the main figures of the manuscript. If you would like to add source data, we would welcome one PDF/Excel-file per figure for this information. These files will be linked online as supplementary "Source Data" files.

IMPORTANT: It is Life Science Alliance policy that if requested, original data images must be made available. Failure to provide original images upon request will result in unavoidable delays in publication. Please ensure that you have access to all original microscopy and blot data images before submitting your revision.

The typical timeframe for revisions is three months. Please note that papers are generally considered through only one revision cycle, so strong support from the referees on the revised version is needed for acceptance.

When submitting the revision, please include a letter addressing the reviewers' comments point by point.

We hope that the comments below will prove constructive as your work progresses. We would be happy to discuss them further once you've had a chance to consider the points raised in this letter.

Thank you for this interesting contribution to Life Science Alliance. We are looking forward to receiving your revised manuscript.

Sincerely,

Andrea Leibfried, PhD
Executive Editor
Life Science Alliance
Meyerhofstr. 1
69117 Heidelberg, Germany
t +49 6221 8891 502
e a.leibfried@life-science-alliance.org
www.life-science-alliance.org

Reviewer #1 (Comments to the Authors (Required)):

This manuscript reports the development of tools to identify the substrates of the arginine methylase CARM1 and to apply these tools to characterize one of them (MED12). In the end, it is a very interesting story of the methylation dependence of the recruitment of a non-coding RNA for gene activation. Although many CARM1 substrates had been described before, this more

comprehensive approach led to entirely new insights regarding the impact of the methylation of one of its substrates. Overall, this manuscript is a no-brainer, but it still requires some clarifications and at least one experiment.

Specific comments:

(1) Abstract: in "CARM1 and its activity" the "its" is ambiguous.
(2) Results, 1st paragraph, last sentence: "The four polyclonal antibodies..." -> since a cocktail of peptides was used, it isn't clear what "four" refers to. Indeed, which one was used for the IP-MS experiments does not seem to be indicated anywhere. Assuming it was all four, this should be explicitly stated.
(3) Fig. 4A: with the labels as shown (and a cryptic legend), this experiment makes no sense at all.
(4) Fig. 4D: there is a strange disconnect between what's claimed in the text and what is actually shown in this figure. There is no evidence whatsoever that R1912 is critical. It wasn't tested by itself and the triple mutant is barely different from the single mutant R1899K.
(5) Description of Fig. 5A: the text says "....CARM1, MED12, and CARM1....". The second CARM1 should probably be H3R17me2a.
(6) Fig. 7A/B: the experiments on the left side of these two graphs seem to be the same (of the same type) and yet, the values and relative binding are remarkably different. Moreover, the legend says nothing about the number of replicates or the statistical analyses.
(7) Fig. 7: a final experiment, which is missing, is to show that the R1899K mutant fails to bind the ncRNA.
(8) ChIP-seq experiments: I could not find any indication about negative controls and replicates. This information must be included.

Reviewer #2 (Comments to the Authors (Required)):

Growing evidence suggests the biological importance of the regulatory role of of arginine side chain methylation. A major regulatory arginine-methyltransferase with broad functionality in signal transfer and chromatin regulation is CARM1, yet knowledge about the range of CARM1 targets is scarce. Because of the diversity in substrate specificity and lack of consensus CARM1 methylation target sites the authors used a mixed methylated peptide immunization strategy to raise antibodies against various known CARM1 methylation sites on several cellular proteins, speculating that these antibodies may also display extended immunoreactivity with other targets because of related but hidden structural similarities/constraints. Affinity purified antibodies obtained from 4 rabbits were then compared for substrate recognition and specificity in WT/KO cells of CARM1 and PRMT1 as a control. Results indicated that 4 antibody preparation may contain novel CARM1 targets, in addition to targets methylated by other PRMTs. IP-MS identified 112 novel CARM1 target proteins, 10 were selected and confirmed as antigens specifically recognized by at least one of the anti-CARM1 methylation specific substrate antibodies, using WT and conditional CARM1 knockdown cells and IP-protein blotting. The authors then focused on the MED12 protein and a CARM1 R-methylation site at position R1899. IP-blotting experiments using WT/mutant MED12 confirmed methylation at R1899 by CARM1 and probably transient interaction with MED12, but no effect on integration of MED12 methylation into the MED complex, suggesting that R1899 is a regulatory site. The authors went on to determine whether known Rme-binding Tudor proteins may interact differentially with a MED12 R1899+/- methylation peptide and identified TDRD3 as a MED12 interacting protein affected by R1899 methylation. Peptide binding and IP of TDRD3 from WT but not from CARM1 knockdown cells co-IPed MED12 and out of three Rme-sites located in the vicinity of R1899 (R1862, R1912), two (R1899, 1892) appear to be critical for MED12-TDRD3

interaction. Genome wide analysis of MED12 and CARM1 is complicated by the cross-reactivity of the available antibodies. Careful comparison of results obtained using different antibody sources nevertheless convincingly revealed canonical ERalpha enhancer sites and G-rich sites as important CARM1 MED12 targets. Four of the ERalpha target genes were further explored and found repressed in CARM1 KO MCF7 but not in WT MCF7 cells. Further, a MED12 MCF7 KO was generated and expression of a ER-target gene panel could be activated with WT but not with R1862/1899/1912 K mutant MED12 constructs. Finally, the MED complex interaction with lnc/ncRNAs was examined by IP/RT-qPCR and members of ncRNA that interact with WT but less with R1899 modification dependent pull-down were identified. Knockdown of one selected ncRNA that maps to the vicinity of the ER target gene GREB1 was shown to suppress GREB1 expression.

The authors provide several sets of convincing data that extend the number of known CARM1 targets. The authors describe in depth a novel connection between CARM1 mediated methylation of MED12 and the regulation of ER target genes in connection to CARM1-MED12 methylation, TDRD3 recruitment and functional connection to the regulatory role of ncRNAs in conjunction with MED12. In addition to an earlier publication where the Bedford lab has revealed cross reactivity between FLAG-antibodies with PRMT5 as a source of misinterpretation of experimental data by other labs they now also show that seemingly H3R17me2a specific antibodies react with a selection of CARM1 target proteins, which may lead to misinterpretation of ChIP data. Although this is just an additional observation, it reflects the careful conduction and interpretation of experimental data. The authors present an important study and I do see only a few shortcomings in the manuscript:

Major:
The mechanistic evidence for the functional CARM1 depending TDRD3-MED12 interaction via MED12 R1899 methylation remains indirect and is currently not entirely convincing, as Figure 4 D still shows TDDR3 binding with the MED12 methylation site mutants (second last and last lanes; R to K mutants # 1899, 1912). Is this due to a technical problem ? Why did the authors not attempt to examine leucine or phenylalanine (up-) mutants of MED12 for compensation of CARM1 deficiency / upregulation of enhancer ncRNAs / ER target gene expression ? Why did they not include / compare genome wide analysis of TDRD3 binding in MED12 and/CARM1 deficient cells ? Although these experimental setups might be difficult to achieve the authors should at least discuss why they omitted such experiments and state that final prove of proposed/implied mechanisms remains an open issue that still needs to be addressed, just in case they can not solve this issue experimentally.

Minor:
1. analysis of CARM1, MED12 ...." the cell type (MCF7) should be mentioned in the text (not only in the Figure Legend).
2. In addition to ER binding sites, SP1 (in the results section and discussion) and C/EBP1 binding sites (discussion only) are mentioned. The SP1 consensus is shown in S3, but information about C/EBP is missing and should be included.

Reviewer #3 (Comments to the Authors (Required)):

Mark Bedford and his colleagues in the manuscript titled "CARM1 methylates MED12 to regulate its RNA binding ability" provides detailed further characterization on the MED12 methylation and its impact on transcription. The biochemical analysis were comprehensive and resourceful, the findings

are truly interesting and significant. This reviewer has only a few questions here.

1. MED12 is a subunit of the Mediator complex which consists of 30 subunits or so in mammalian cells, however, MED12 seems to be treated as a single protein in this study. It is not clear whether methylation on MED12 affects functions of MED12 alone or the whole complex throughout the whole manuscript.
2. Since MED12 is a subunit within the CDK8 kinase submodule, does MED12 methylation affects the CDK8 kinase submodule structure and functions, especially the kinase activity? When MED12 was knockout, what happened to the rest of Mediator complex?
3. It is an important finding that MED12/ncRNA interaction is dependent on MED12 methylation by CARM1. A previous finding (Lai et al. 2013) seemed to demonstrate that disease-related mutations within the MED12 subunit disrupts the interaction between MED12/ncRNA. One wonders if there is any connection between these findings.
4. Figure 4A panel seems to have mistake in labeling.

Point-by-point rebuttal

We would like to thank the reviewers for there constructive criticism. As a result of the reviewer's suggestions and requests the manuscript is substantially improved.

Reviewer #1:

Overall, this manuscript is a no-brainer, but it still requires some clarifications and at least one experiment.

Specific comments:

(1) Abstract: in "CARM1 and its activity" the "its" is ambiguous.

To clarify, we have changed "its activity" to "the methyl mark it deposits.

(2) Results, 1st paragraph, last sentence: "The four polyclonal antibodies..." -> since a cocktail of peptides was used, it isn't clear what "four" refers to. Indeed, which one was used for the IP-MS experiments does not seem to be indicated anywhere. Assuming it was all four, this should be explicitly stated.

To remove any confusion, we added "using a cocktail of the four ADMA^{CARM1} antibodies" on page 7 in the first sentence of the second paragraph, which talks about the IP-MS experiment.

(3) Fig. 4A: with the labels as shown (and a cryptic legend), this experiment makes no sense at all.

This is indeed a mistake. Our (+) and (-) signs were scrambled at some point during the figure assembly. It is now clear that the unmethylated peptide can act as a substrate for recombinant CARM1, but the methylated peptide cannot because the methyl acceptor site is already occupied.

(4) Fig. 4D: there is a strange disconnect between what's claimed in the text and what is actually shown in this figure. There is no evidence whatsoever that R1912 is critical. It wasn't tested by itself and the triple mutant is barely different from the single mutant R1899K.

We agree with the reviewer on this point and have changed to wording as follows: "These co-immunoprecipitation experiments revealed that the R1862 and R1912 are not critical for this interaction, but that the R1899 is important."

(5) Description of Fig. 5A: the text says "....CARM1, MED12, and CARM1....". The second CARM1 should probably be H3R17me2a.

We have changed “CARM1 activity” to “H3R17me2a”.

(6) Fig. 7A/B: the experiments on the left side of these two graphs seem to be the same (of the same type) and yet, the values and relative binding are remarkably different.

We thank the review for his/her keen eye. There are two “issues” with Figure 7A/B.

<u>First</u>, we had listed MCF-7 as the control line in the A and B panels. This is not totally correct, because they actually represent stable selected MCF-7 lines that harbor the Doc-inducible knockdown vectors for CARM1 (A) and TDRD3 (B). They are thus different lines that were both derived from MCF7 cells, and we have re-labeled the figure to highlight this difference. This may also explain why there are slight differences between the relative binding patterns in the controls for panel A and B.

<u>Second</u>, after viewing our data, we found that we had used different pair of primers for ncRNA-a7 in panel A and B. The Nature manuscript from the Shiekhattar lab that describes ncRNA-a7 used two different primer sets to detect this RNA by RT-qPCR. The bars for ncRNA-a7 in Fig. 7A came from data using primer1, and the bars of ncRNA-a7 in Fig.7B and Fig.S6A/B came from data using primer2. Now we used primer2 for all RIP experiments. The data in Fig. 7A & B can now be compared more accurately. The primer information in the Supplementary Table 1 is correct.

Moreover, the legend says nothing about the number of replicates or the statistical analyses.

To address this issue we have now added the following sentence in the Figure 7A/B legend “Error bars represent standard deviation based on replicates (n=3)”. We have also added a new “Statistical Analysis” section to the “Materials and Methods” to address the statistical analysis that was used for all the qPCR experiments.

(7) Fig. 7: a final experiment, which is missing, is to show that the R1899K mutant fails to bind the ncRNA.

This is indeed a critical experiment. We had actually performed this experiment, but it was presented in the Supplementary Figure section (S6B) of the first version of this manuscript, and not as part of a primary figure. We have now moved it into the main Figure 7, as new panel C.

(8) ChIP-seq experiments: I could not find any indication about negative controls and replicates. This information must be included.

> *For the ChIP-seq experiments the negative controls are input DNA. This information is now provided in the “Peak Calling and Gene Annotation” section in the “Materials and Methods”. There are no replicates.*

Reviewer #2:

The authors present an important study and I do see only a few shortcomings in the manuscript:

Major:
The mechanistic evidence for the functional CARM1 depending TDRD3-MED12 interaction via MED12 R1899 methylation remains indirect and is currently not entirely convincing, as Figure 4 D still shows TDDR3 binding with the MED12 methylation site mutants (second last and last lanes; R to K mutants # 1899, 1912). Is this due to a technical problem?

> *We agree that there is still a weak interaction between MED12 and TDRD3 even in the triple mutant. We think that MED12 may have additional CARM1 methylation sites that could interact weakly with TDRD3. What is clear is that the MED12 R1899me2a site is the primary binding site for TDRD3.*

Why did the authors not attempt to examine leucine or phenylalanine (up-) mutants of MED12 for compensation of CARM1 deficiency / upregulation of enhancer ncRNAs / ER target gene expression?

> *The notion of whether or not the phenylalanine mutation accurately mimics arginine methylation is the subject of debate. Phenylalanine only mimicked the hydrophobic characteristic of methylated arginine by not positive charge. Only a few published papers have used this mimic replacement approach, and none have presented evidence that this replacement will facilitate an interaction with the aromatic cage of a Tudor domain.*

Why did they not include / compare genome wide analysis of TDRD3 binding in MED12 and/CARM1 deficient cells? Although these experimental setups might be difficult to achieve the authors should at least discuss why they omitted such experiments and state that final prove of proposed/implied mechanisms remains an open issue that still needs to be addressed, just in case they can not solve this issue experimentally.

> *This is an important suggestion, and we are currently planning to perform TDRD3 ChIP-seq analysis in MED12 and CARM1 deficient cells as the fellow–up story.*

Minor:
1. Analysis of CARM1, MED12 ...." the cell type (MCF7) should be mentioned in the text (not only in the Figure Legend).

We added MCF-7 cells in the text.

2. In addition to ER binding sites, SP1 (in the results section and discussion) and C/EBP1 binding sites (discussion only) are mentioned. The SP1 consensus is shown in S3, but information about C/EBP is missing and should be included.

The reason we have mentioned the C/EBP consensus-binding motif only in the discussion section is because it was identified in Joe Torchia's paper. In our study, the C/EBP motif did not display any enrichment. We have now clarified this issue by explicitly state this in the discussion section – "We did not observe enrichment for the C/EBPα motif in our ChIP-seq experiment".

Reviewer #3:

The biochemical analysis were comprehensive and resourceful, the findings are truly interesting and significant. This reviewer has only a few questions here.

1. MED12 is a subunit of the Mediator complex, which consists of 30 subunits or so in mammalian cells, however, MED12 seems to be treated as a single protein in this study. It is not clear whether methylation on MED12 affects functions of MED12 alone or the whole complex throughout the whole manuscript.

We think that methylation of MED12 impacts the function of this subunit of the Mediator complex, in the context of the complex. We have no evidence that methylation of the R1899 site of MED12 has any Mediator independent functions.

2. Since MED12 is a subunit within the CDK8 kinase submodule, does MED12 methylation affects the CDK8 kinase submodule structure and functions, especially the kinase activity? When MED12 was knockout, what happened to the rest of Mediator complex?

This is an important experiment, and we have tried unsuccessfully to fully address this issue. We performed in vitro kinase assays with the MED12 IPed from CARM1 WT and KO cells (which presumably pulls-down CDK8), we did not observe altered kinase activity between these two samples. However, we did not include this data because our control CDK8 IP sample showed the same degree of histone H3 phosphorylation, even though there was much more CDK8 protein in this sample. We think that this experiment was performed with an excess of CDK8 and that the histone was being fully phosphorylated in all the samples. We need to better control this experiment in the future.

Regarding Mediator minus MED12; MED13, which links the entire kinase complex to core Mediator, will remain bound in the absence of MED12. Depletion of MED12, which links CycC-CDK8/19 to MED13, will lead to loss of CycC-CDK8/19. However, the MED12 paralog MED12L, should it be expressed in the cell type concerned, could substitute for MED12 at least partially and retain CycC-CDK8/19 in Mediator, diminishing the effect of MED12 depletion. Importantly, the MED12 arginine residues that are methylated by CARM1 are not present in MED12L.

3. It is an important finding that MED12/ncRNA interaction is dependent on MED12 methylation by CARM1. A previous finding (Lai et al. 2013) seemed to demonstrate that disease-related mutations within the MED12 subunit disrupts the interaction between MED12/ncRNA. One wonders if there is any connection between these findings.

R1899 mutations have not been identified diseases. We have not tested the reported Lai et al. mutations, which disrupts the interaction between MED12/ncRNA, to see if they some how impact the interaction between MED12 and TDRD3, but this would be interesting. We will do so in the future.

4. Figure 4A panel seems to have mistake in labeling.

This is indeed a mistake. Our (+) and (-) signs were scrambled at some point during the figure assembly. We have corrected this error. It is now clear that the unmethylated peptide can act as a substrate for recombinant CARM1, but the methylated peptide cannot because the methyl acceptor site is already occupied.

September 3, 2018

RE: Life Science Alliance Manuscript #LSA-2018-00117-TR

Dr. Mark T. Bedford
MD Anderson Cancer Center
Epigenetics and Molecular Carcinogenesis
Science Park
Park road 1C
Smithville, Texas 78957

Dear Dr. Bedford,

Thank you for submitting your revised manuscript entitled "CARM1 Methylates MED12 to Regulate its RNA Binding Ability". As you will see, the reviewers appreciate the introduced changes, and we would be happy to publish your paper in Life Science Alliance pending final revisions:

Please provide the Suppl Tables (missing from submission) and make sure that the tables included in Figures 1 and 2 are of production quality. Please also check one more time whether the number of experiments performed for each dataset are indicated everywhere.

To upload the final version of your manuscript, please log in to your account:
https://lsa.msubmit.net/cgi-bin/main.plex
You will be guided to complete the submission of your revised manuscript and to fill in all necessary information.

To avoid unnecessary delays in the acceptance and publication of your paper, please read the following information carefully.

A. FINAL FILES:

These items are required for acceptance.

-- An editable version of the final text (.DOC or .DOCX) is needed for copyediting (no PDFs).

-- High-resolution figure, supplementary figure and video files uploaded as individual files: See our detailed guidelines for preparing your production-ready images, http://life-science-alliance.org/authorguide

-- Summary blurb (enter in submission system): A short text summarizing in a single sentence the study (max. 200 characters including spaces). This text is used in conjunction with the titles of papers, hence should be informative and complementary to the title. It should describe the context and significance of the findings for a general readership; it should be written in the present tense and refer to the work in the third person. Author names should not be mentioned.

B. MANUSCRIPT ORGANIZATION AND FORMATTING:

Full guidelines are available on our Instructions for Authors page, http://life-science-alliance.org/authorguide

We encourage our authors to provide original source data, particularly uncropped/-processed electrophoretic blots and spreadsheets for the main figures of the manuscript. If you would like to add source data, we would welcome one PDF/Excel-file per figure for this information. These files will be linked online as supplementary "Source Data" files.

Submission of a paper that does not conform to Life Science Alliance guidelines will delay the acceptance of your manuscript.

It is Life Science Alliance policy that if requested, original data images must be made available to the editors. Failure to provide original images upon request will result in unavoidable delays in publication. Please ensure that you have access to all original data images prior to final submission.

The license to publish form must be signed before your manuscript can be sent to production. A link to the electronic license to publish form will be sent to the corresponding author only. Please take a moment to check your funder requirements.

Reviews, decision letters, and point-by-point responses associated with peer-review at Life Science Alliance will be published online, alongside the manuscript. If you do want to opt out of this transparent process, please let us know immediately.

Thank you for your attention to these final processing requirements. Please revise and format the manuscript and upload materials within 7 days.

Thank you for this interesting contribution, we look forward to publishing your paper in Life Science Alliance.

Sincerely,

Andrea Leibfried, PhD
Executive Editor
Life Science Alliance
Meyerhofstr. 1
69117 Heidelberg, Germany
t +49 6221 8891 502
e a.leibfried@life-science-alliance.org
www.life-science-alliance.org

Reviewer #1 (Comments to the Authors (Required)):

The authors have adequately addressed my concerns and I believe also those of the other reviewers. The additional experiment I had asked for had been hiding in the supplementary (and has now been promoted).

Reviewer #2 (Comments to the Authors (Required)):

The authors have dealt with all points and issues previously raised by the reviewers and completed all necessary corrections in the text and figures.

September 7, 2018

RE: Life Science Alliance Manuscript #LSA-2018-00117-TRR

Dr. Mark T. Bedford
MD Anderson Cancer Center
Epigenetics and Molecular Carcinogenesis
Science Park
Park road 1C
Smithville, Texas 78957

Dear Dr. Bedford,

Thank you for submitting your Research Article entitled "CARM1 Methylates MED12 to Regulate its RNA Binding Ability". It is a pleasure to let you know that your manuscript is now accepted for publication in Life Science Alliance. Congratulations on this interesting work.

The final published version of your manuscript will be deposited by us to PubMed Central (PMC) as soon as we are allowed to do so, the application for PMC indexing has been filed. You may be eligible to also deposit your Life Science Alliance article in PMC or PMC Europe yourself, which will then allow others to find out about your work by Pubmed searches right away. Such author-initiated deposition is possible/mandated for work funded by eg NIH, HHMI, ERC, MRC, Cancer Research UK, Telethon, EMBL.
Please also see:
https://www.ncbi.nlm.nih.gov/pmc/about/authorms/
https://europepmc.org/Help#howsubsmanu

Your manuscript will now progress through copyediting and proofing. It is journal policy that authors provide original data upon request.

Reviews, decision letters, and point-by-point responses associated with peer-review at Life Science Alliance will be published online, alongside the manuscript. If you do want to opt out of this transparent process, please let us know immediately.

IMPORTANT: If you will be unreachable at any time, please provide us with the email address of an alternate author. Failure to respond to routine queries may lead to unavoidable delays in publication.

Scheduling details will be available from our production department. You will receive proofs shortly before the publication date. Only essential corrections can be made at the proof stage so if there are any minor final changes you wish to make to the manuscript, please let the journal office know now.

DISTRIBUTION OF MATERIALS:
Authors are required to distribute freely any materials used in experiments published in Life Science Alliance. Authors are encouraged to deposit materials used in their studies to the appropriate repositories for distribution to researchers.

You can contact the journal office with any questions, contact@life-science-alliance.org

Again, congratulations on a very nice paper. I hope you found the review process to be constructive and are pleased with how the manuscript was handled editorially. We look forward to future exciting submissions from your lab.

Sincerely,

Andrea Leibfried, PhD
Executive Editor
Life Science Alliance
Meyerhofstr. 1
69117 Heidelberg, Germany
t +49 6221 8891 502
e a.leibfried@life-science-alliance.org
www.life-science-alliance.org
